# Global prevalence of macrolide-resistant *Staphylococcus* spp.: a comprehensive systematic review and meta-analysis

**DOI:** 10.3389/fmicb.2025.1524452

**Published:** 2025-03-14

**Authors:** Tahereh Navidifar, Abbas Zare Banadkouki, Elnaz Parvizi, Maryam Mofid, Narges Golab, Masoumeh Beig, Mohammad Sholeh

**Affiliations:** ^1^Department of Basic Sciences, Shoushtar Faculty of Medical Sciences, Shoushtar, Iran; ^2^Department of Microbiology, Shahid Beheshti University, Tehran, Iran; ^3^Quality Control Department of Temad Mfg, Co., Tehran, Iran; ^4^Department of Microbiology, Science and Research Branch, Islamic Azad University, Tehran, Iran; ^5^School of Medicine, Hamadan University of Medical Sciences, Hamadan, Iran; ^6^Department of Microbiology, School of Medicine, Tehran University of Medical Sciences, Tehran, Iran; ^7^Department of Bacteriology, Pasteur Institute of Iran, Tehran, Iran

**Keywords:** *Staphylococcus*, macrolide, meta-analysis, methicillin-resistant *Staphylococcus aureus*, coagulase-negative staphylococci

## Abstract

**Background:**

*Staphylococcus* is a genus of bacteria responsible for various infections ranging from mild skin to severe systemic diseases. Methicillin-resistant *Staphylococcus aureus* (MRSA) and coagulase-negative staphylococci (CoNS) are significant challenges owing to their resistance to multiple antibiotics, including macrolides, such as erythromycin, clarithromycin, and azithromycin.

**Objective:**

This study aimed to systematically review and synthesize data on the prevalence of macrolide resistance in *Staphylococcus* spp., identify trends and changes in resistance patterns over time, and assess how testing methods and guidelines affect reported resistance rates.

**Methods:**

The study conducted a systematic search of the Scopus, PubMed, Web of Science, and EMBASE databases. Studies have reported the proportion of macrolide-resistant *Staphylococcus* spp. Two authors independently extracted and analyzed the data using a random-effects model. Heterogeneity was assessed, and subgroup analyses were performed based on country, continent, species, AST guidelines, methods, and period.

**Results:**

In total, 223 studies from 76 countries were included. The pooled prevalence of resistance to erythromycin, clarithromycin, and azithromycin were 57.3, 52.6, and 57.9%, respectively. Significant heterogeneity was observed across studies (I^2^ > 95%, *p* < 0.001). Oceania (72%) had the highest erythromycin resistance, whereas Europe had the lowest (40.7%). Subgroup analyses revealed variations in resistance based on the species, with higher resistance in MRSA than in MSSA and CoNS than in other species. Over time, a slight decrease in erythromycin resistance has been observed (59.6% from 2015–2019 to 55% from 2020–2023).

**Conclusion:**

This study emphasizes the high prevalence of macrolide resistance in *Staphylococcus* spp. and its notable regional variation. These findings highlight the necessity for standardized methodologies and global surveillance to manage macrolide resistance effectively. Controlling antibiotic resistance should prioritize enhancing public health measures and updating treatment guidelines.

**Systematic review registration:**

https://www.crd.york.ac.uk/prospero/display_record.php?RecordID=557756, CRD42024557756.

## 1 Introduction

*Staphylococcus* is a genus of bacteria that can cause many infections, from mild skin infections to serious systemic diseases. These infections can affect the skin, lungs, bloodstream, and medical devices and have become a significant treatment challenge, particularly for methicillin-resistant *Staphylococcus aureus* (MRSA) (Tong et al., 2015; Cheung et al., 2021). It is estimated that approximately 30% of people carry *S. aureus* on their bodies without any symptoms. In 2019, *S. aureus* was associated with more than 1 million deaths, with an estimated range of 816,000 to 1,470,000 deaths (Ikuta et al., 2022). In the United States, the rate of invasive MRSA infections in the black population (66.5 cases per 100,000 person-years) is more than twice that of the white population (27.7 cases per 100,000 person-years). In Australia, the incidence of *Staphylococcus aureus* bacteremia (SAB) is 5.8 to 20 times higher among Indigenous Australians than among non-Indigenous Australians. Similarly, in New Zealand, Māori and Pacific Island communities have significantly higher rates of SAB than those of European descent (Tong et al., 2015). In recent years, there has been a significant increase in the rate of MRSA colonization in healthy individuals, potentially contributing to the spread of MRSA in both community and hospital settings (Barcudi et al., 2020). In addition, MRSA is a pathogen resistant to multiple antibiotics, complicating infection management and leading to increased healthcare costs and adverse outcomes (Abebe and Birhanu, 2023; Lan et al., 2024; Saleem et al., 2025). Globally, the pathogen-drug combination with the most significant increase in attributable burden was MRSA. Its attributable deaths have doubled from 57,200 (range 34,100-80,300) in 1990 to 130,000 (range 113,000-146,000) in 2021(Naghavi et al., 2024).

Antibiotic resistance is a global health crisis that threatens the effectiveness of treatments for bacterial infections. Misuse and overuse of antibiotics have accelerated the development of resistance, rendering many therapies ineffective (Yadav and Kapley, 2021; Estany-Gestal et al., 2024). Macrolides, such as erythromycin, clarithromycin, and azithromycin, are widely used to treat various staphylococcal infections. However, the increasing emergence of macrolide resistance in *Staphylococcus* spp. has become a critical challenge in treating infections caused by these bacteria. Resistance to macrolides has been attributed to the methylation of specific targets in the 23S rRNA by methylases encoded by *erm* genes, particularly *erm*(C) and *erm*(A), which can be constitutive or inducible. In addition, efflux pumps, such as ABC-F proteins encoded by *msr* genes and major facilitator superfamily transporters encoded by *mef* genes, drug inactivation by phosphotransferases encoded by *mph* genes, and esterase encoded by *ere* genes, confer macrolide resistance (Leclercq, 2002; Miklasinska-Majdanik, 2021; El Mammery et al., 2023; Mahfouz et al., 2023). These mechanisms show regional variation, reflecting differences in the prevalence of resistance genes and differences in antibiotic use practices (Miklasinska-Majdanik, 2021).

Overall, antibiotic resistance reduces the effectiveness of these antibiotics and complicates the treatment of common staphylococcal infections such as skin infections, pneumonia, and bacteremia.

The global burden of macrolide-resistant staphylococci affects both public health and healthcare systems. Data indicate increasing infection rates and resistance patterns, particularly in healthcare-associated infections where *S. aureus* is a leading cause of morbidity and mortality (An et al., 2024). The economic impact is also profound, with resistant infections leading to longer hospital stays, more complex treatment regimens, and increased healthcare costs (Lodise and McKinnon, 2007). However, the limited number of effective treatment options for resistant infections increases the risk of adverse outcomes. This underscores the importance of developing novel therapeutic approaches and implementing stringent infection control measures (Guo et al., 2020).

Previous research on macrolide resistance in *staphylococci* has been limited by study design and reporting inconsistencies, making it difficult to draw robust conclusions and identify consistent trends. In addition, many studies require extensive regional analyses, limiting the generalizability of findings and their impact on global health. Furthermore, gaps in understanding the temporal trends and dynamics of resistance highlight the need for longitudinal studies and broader surveillance efforts (Leclercq, 2002; Khader et al., 2019). Hence, standardized methodologies and collaborative efforts across regions are essential to improving our understanding and managing macrolide resistance in *staphylococci*.

The primary objective of this study was to systematically review and analyze the available data on the prevalence of macrolide resistance in *Staphylococcus* spp.

The secondary objectives were to identify trends and changes in resistance patterns over time, explore heterogeneity in resistance rates across regions and populations, and assess the impact of testing methods and guidelines on reported resistance rates. By addressing these objectives, this study aimed to fill the existing knowledge gaps and provide comprehensive insights into the dynamics of macrolide resistance in *Staphylococcus* spp. to guide future research and clinical practice.

## 2 Methods

This study was conducted according to PRISMA guidelines and included a meta-analysis to increase the robustness of the results. The study was registered in the PROSPERO registry under the code CRD42024557756.

### 2.1 Eligibility criteria

The inclusion criteria for this meta-analysis stipulated that studies must investigate *Staphylococcus* spp. macrolide resistance, report resistance rates, specify sample size determination and have complete English-language articles available. Only cross-sectional studies providing antimicrobial resistance (AMR) data, mainly those reporting baseline resistance levels before any interventions, were included. Such studies offer a population-based overview of resistance rates at a specific time and are, therefore, suitable for estimating the prevalence of macrolide resistance. Studies were excluded if published in languages other than English and were review articles, case reports, and case series studies.

### 2.2 Information sources

A comprehensive search was conducted in several major online databases, including Scopus, PubMed, Web of Science, and EMBASE, focusing on studies published through December 2023. These databases were selected for their extensive coverage of biomedical literature, ensuring a broad scope for the systematic review.

### 2.3 Search strategy

The search syntax was tailored to each database according to their respective guidelines (“Staphylococcus*” OR “*S. aureus*” OR “*S. epidermidis*” OR “*S. saprophyticus*” OR “*S. lugdunensis*” OR “*S. hominis*” OR “*S. capitis*” OR “*S. haemolyticus*” OR “CoNS” OR “MRCoNS” OR “MRSA” OR “MSCoNS” OR “VISA” OR “VSSA”) AND (macrolide* OR azithromycin OR clarithromycin OR erythromycin OR roxithromycin OR telithromycin OR spiramycin OR fidaxomicin) AND (resistant* OR susceptible*). This rigorous methodological approach ensured comprehensive coverage of relevant research topics.

### 2.4 Selection process

The systematic online database search results were imported into EndNote (version 20), removing duplicate entries. Two authors (NG and EP) independently screened and analyzed the relevant publications to minimize bias. Disagreements were resolved by a third author (TN).

### 2.5 Data collection process

Data extracted included first author(s), publication year, country, diagnostic method, sample source, number of positive tests, and total sample size. To ensure accuracy, two authors (MM and MB) extracted the data independently, and any disagreements were resolved by consensus.

### 2.6 Study risk of bias assessment

The quality of the included studies was assessed using the JBI tool. Two authors (MB and TN) independently evaluated the quality, and a third author (MSH) resolved disagreements.

### 2.7 Synthesis methods

This comprehensive systematic review and meta-analysis aimed to determine the global prevalence of macrolide-resistant *Staphylococcus* species. The analysis used proportions as the primary outcome measure. The main objective was to assess the prevalence of macrolide-resistant *Staphylococcus* strains, while the secondary objective sought to identify sources of heterogeneity between studies. Subgroup analyses investigated potential variability in resistance rates across different demographic and methodological factors. Additionally, trends in macrolide resistance over time were examined.

A random effects model was employed to analyze the data, allowing for considering variability within and between studies. The degree of heterogeneity was estimated using the DerSimonian-Laird method for τ^2^. Along with τ^2^, the Q-test for heterogeneity and the I^2^ statistic (Higgins and Thompson, 2002) were also calculated. Heterogeneity was considered present if τ^2^ > 0, regardless of the Q-test results.

Subgroup analyses were performed across various factors to explore sources of heterogeneity, including countries, continents, antibiotic susceptibility testing (AST) guidelines, AST methods, *Staphylococcus* species, coagulase status, and year groups. This stratification helped identify macrolide resistance patterns and potential drivers across regions and testing protocols.

A Logit Transformation was applied to the proportions of macrolide-resistant *Staphylococcus* species to account for variations in the proportion data and stabilize the variance. The logit transformation—also known as the log-odds transformation—was used to ensure that the outcome variable remained within the 0 to 1 range, mainly when dealing with extreme proportions of resistance. This transformation also normalized the distribution of proportions, facilitating more accurate meta-regression modeling.

Meta-regression analysis was conducted to explore temporal trends in macrolide resistance over time. Moderator variables included country, continent, AST guidelines, and year group. This analysis aimed to identify how macrolide resistance in *Staphylococcus* species has evolved across different geographical regions and under varying testing conditions.

Outliers and influential studies were identified using studentized residuals and Cook’s distances. Studies with studentized residuals exceeding the 100 × (1–0.05 / (2 × k)) th percentile of a standard normal distribution were flagged as potential outliers (after applying a Bonferroni correction for *α* = 0.05 and for k studies in the meta-analysis). Studies with Cook’s distances greater than the median plus six times the interquartile range of Cook’s distances were considered influential and examined for their impact on the overall estimates.

Funnel plot asymmetry was assessed using rank correlation and regression tests, with the standard error of the observed results serving as the predictor. This approach was used to evaluate potential publication bias. All statistical analyses were performed using R (version 4.2.1) and the metafor package (version 3.8.1) (Cochran, 1954; Begg and Mazumdar, 1994; Higgins and Thompson, 2002; Sterne and Egger, 2005; Viechtbauer, 2010; Viechtbauer and Cheung, 2010; Kuhn et al., 2015).

## 3 Results

### 3.1 Descriptive statistics

**Figure 1:**
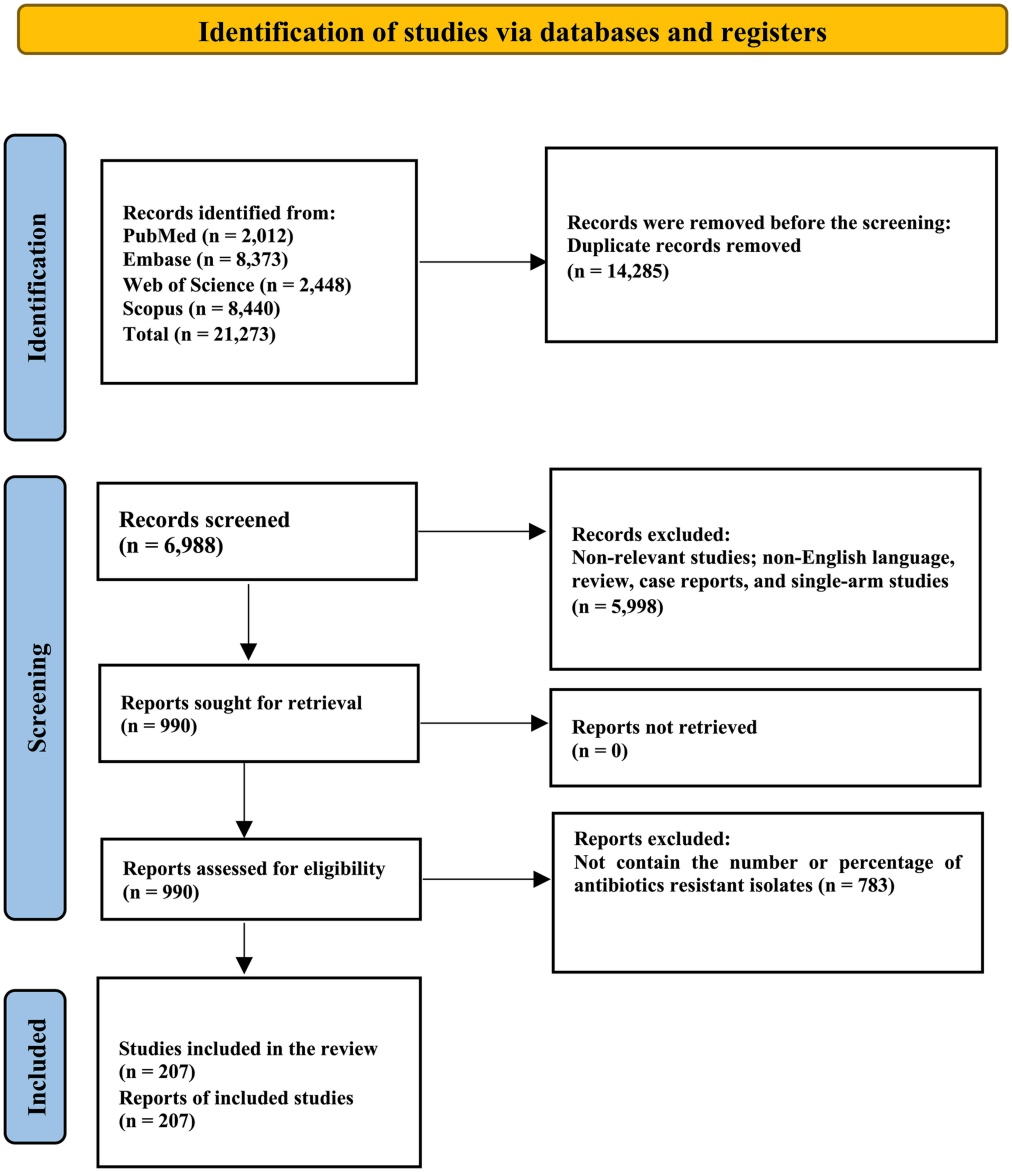
PRISMA flow diagram of study selection: this diagram illustrates the process of study identification, screening, eligibility assessment, and inclusion for the review. From a total of 21,273 records identified through databases, 207 studies were included in the final review after exclusion based on criteria such as duplication, irrelevance, and lack of data on antibiotic-resistant isolates.

A total of 21,273 records as results of the systematic search were collected in reference manager software (EndNote version 20), and 14,285 duplicated articles were removed. Thousand eighty-eight articles were assessed in the title abstract for this section; 990 full-text articles were evaluated and excluded. Eventually, this systematic review and meta-analysis included 207 eligible studies. The reports came from 76 countries and six continents. The reports cover the years 2015 to 2023. The screening and selection of presages are summarized in the PRISMA flowchart (Figure 1). Characteristics and references of included studies are presented in Table 1.

**Table 1:** A summary of the included studies in the meta-analysis is provided below, highlighting the characteristics employed.

Author	Countries	AST method	AST guideline	Quality group	Species	Erythromycin	Clarithromycin	Azithromycin
Asbell et al. (2015)	United States	MM	C	L	MRSA	ND	ND	283
Abbasi et al. (2017)	Iran	DD	C	L	MRSA	30	ND	ND
Changchien et al. (2016)	China	DD	C	L	MRSA	159	ND	ND
Qin et al. (2017)	China	MIC	C	L	MRSA	109	ND	ND
Baek et al. (2016)	South Korea	AM	C	L	MRSA	338	ND	ND
Noordin et al. (2016)	Malaysia	DD	C	L	MRSA	297	ND	ND
Gitau et al. (2018)	Kenya	DD	C	L	MRSA	129	ND	ND
Coombs et al. (2020)	Australia	AM	C	L	MRSA	174	ND	ND
Shashindran et al. (2016)	ND	DD	C	L	MRSA	84	ND	ND
Horvath et al. (2020)	Hungary	MM	E	L	MRSA	122	ND	ND
Numanovic et al. (2021)	ND	DD	E	L	MRSA	9	ND	ND
Nichol et al. (2019)	Canada	MM	C	L	MRSA	ND	305	ND
Chaleshtori and Kachoie (2016)	ND	DD	C	L	MRSA	ND	ND	10
Chen Y. L. et al. (2021)	Taiwan	DD	C	L	MRSA	16	ND	ND
Khemiri et al. (2017)	Libya	DD	E	L	MRSA	30	ND	ND
Li et al. (2016)	China	MIC	C	L	MRSA	553	ND	ND
Napp et al. (2016)	United States	ND	ND	L	MRSA	ND	ND	37
Akbariyeh et al. (2017)	ND	DD	C	S	MRSA	2	ND	ND
Elzorkany et al. (2019)	India	DD	C	L	MRSA	159	ND	ND
Dormanesh et al. (2015)	Iran	DD	C	L	MRSA	32	ND	ND
Larsen et al. (2015)	Denmark	DD	E	L	MRSA	56	ND	ND
Valle et al. (2016)	Philippines	AM	C	L	MRSA	3	ND	ND
Guo et al. (2021)	China	DD	C	L	MRSA	65	ND	ND
Tekeli et al. (2016)	Turkey	AM	C	L	MRSA	131	ND	ND
Xie et al. (2016)	China	DD	C	L	MRSA	58	ND	ND
Nasirian et al. (2018)	Iran	DD	C	L	MRSA	88	ND	ND
Chauhan et al. (2021)	India	DD	C	H	MRSA	15	ND	ND
Livermore et al. (2015)	ND	MM	E	L	MRSA	123	ND	ND
Modukuru et al. (2021)	India	DD	C	H	MRSA	174	ND	ND
Ukpai et al. (2021)	Nigeria	DD	MG	L	MRSA	122	ND	ND
Pushkar et al. (2022)	India	DD	C	L	MRSA	31	ND	ND
Islam and Shamsuzzaman (2015)	Bangladesh	DD	C	L	MRSA	ND	ND	11
Preeja et al. (2021)	India	DD	C	L	MRSA	54	ND	ND
Yao et al. (2023)	China	AM	C	L	MRSA	173	ND	ND
Conceicao et al. (2021)	Portugal	DD	E	L	MRSA	92	ND	ND
Raut et al. (2017)	Nepal	DD	C	L	MRSA	40	ND	ND
Pradhan et al. (2021)	Nepal	DD	C	L	MRSA	964	ND	ND
El-Baghdady et al. (2020)	Egypt	DD	C	L	MRSA	94	ND	ND
Liang et al. (2018)	China	AM	C	L	MRSA	51	ND	ND
Fateh Amirkhiz et al. (2015)	Iran	DD	C	L	MRSA	ND	ND	30
Chen P. Y. et al. (2021)	Taiwan	MM	C	L	MRSA	233	ND	ND
Taherirad et al. (2016)	Iran	DD	C	L	MRSA	36	ND	ND
Bhattacharya et al. (2016)	India	DD	C	L	MRSA	ND	180	ND
Ukpai et al. (2021)	Nigeria	DD	C	L	MRSA	122	ND	ND
Leibler et al. (2017)	United States	AM	C	L	MRSA	13	ND	ND
Lee et al. (2020)	Taiwan	MIC	C	L	MRSA	889	ND	ND
Kong et al. (2018)	China	DD	C	L	MRSA	5	ND	ND
Petrović et al. (2016)	Serbia	DD	C	L	MRSA	27	ND	ND
de Benito et al. (2018)	Spain	DD	C	L	MRSA	45	ND	ND
Goudarzi et al. (2018)	Iran	DD	C	L	MRSA	50	ND	ND
Ouidri (2018)	Algeria	DD	C	L	MRSA	9	ND	ND
Esmaeili Benvidi et al. (2017)	Iran	DD	C	L	MRSA	59	ND	ND
Yitayeh et al. (2021)	Ethiopia	DD	C	L	*S. Saprophiticus*	12	ND	ND
Asbell et al. (2015)	United States	MM	C	L	MRCONS	ND	ND	120
Sheeba et al. (2021)	India	ND	C	L	CONS	182	ND	ND
Almasri et al. (2016)	Palestinian Territories	MIC	C	L	*Staphylococcus Spp*	131	ND	ND
Maleki et al. (2019)	Iran	DD	C	L	*S. aureus*	18	ND	ND
Peng et al. (2021)	China	AM	C	L	*S. haemolyticus*	35	ND	ND
Al-Naqshbandi et al. (2019)	Iraq	AM	ND	L	*S. haemolyticus*	30	ND	ND
Pfaller et al. (2020)	ND	MM	E	L	*S. haemolyticus*	159	ND	ND
Bensaci and Sahm (2017)	United States	MM	E	L	*S. haemolyticus*	406	ND	ND
Khan et al. (2017)	Qatar	AM	C	L	*S. haemolyticus*	19	ND	ND
Khan et al. (2017)	India	DD	C	L	*S. haemolyticus*	4	ND	ND
Murugesan et al. (2015)	India	DD	C	L	*S. haemolyticus*	9	ND	ND
Bolatchiev (2020)	Russia	DD	E	L	*S. haemolyticus*	19	ND	ND
Belbase et al. (2017)	Nepal	DD	C	L	*S. haemolyticus*	34	ND	ND
Junaidi et al. (2023)	Malaysia	DD	C	L	*S. haemolyticus*	53	ND	61
Zamanian et al. (2021)	Iran	DD	C	L	*S. haemolyticus*	1,010	ND	ND
Ackers-Johnson et al. (2021)	Uganda	DD	E	L	*S. haemolyticus*	14	ND	ND
Kang and Kim (2019)	South Korea	ND	ND	L	*S. haemolyticus*	10	ND	ND
Al-Habsi et al. (2020)	Oman	AM	C	L	*S. haemolyticus*	2	ND	ND
Skender et al. (2022)	India	MIC	C	L	*S. haemolyticus*	1	ND	ND
Solomon and Salaudeen (2021)	Nigeria	DD	C	L	*S. haemolyticus*	7	ND	ND
Saxena et al. (2019)	India	DD	C	L	*S. haemolyticus*	3	ND	ND
Xu et al. (2019)	China	DD	C	L	*S. haemolyticus*	2	ND	ND
Talapan et al. (2023)	Romania	MM	C	L	*S. haemolyticus*	835	ND	ND
Cavanagh et al. (2016)	Norway	MIC	E	L	*S. haemolyticus*	29	ND	ND
Guo et al. (2019)	China	DD	C	L	*S. haemolyticus*	184	ND	ND
Shittu et al. (2015)	Nigeria	DD	C	L	*S. haemolyticus*	10	ND	ND
Bishr et al. (2021)	Egypt	MM	C	L	*S. haemolyticus*	19	ND	18
Getaneh et al. (2021)	Ethiopia	DD	C	L	*S. haemolyticus*	30	ND	ND
Mutonga et al. (2019)	Kenya	AM	ND	L	*S. haemolyticus*	3	ND	ND
Kumar et al. (2018)	India	ND	C	L	*S. haemolyticus*	29	ND	ND
Belete (2020)	Ethiopia	DD	C	L	*S. haemolyticus*	7	ND	ND
Bhavana et al. (2019)	India	DD	C	L	*S. haemolyticus*	6	ND	ND
Peterside et al. (2015)	Nigeria	DD	C	L	*S. haemolyticus*	27	ND	ND
Al-Taweel (2020)	Iraq	DD	C	L	*S. haemolyticus*	9	ND	ND
Hasanvand et al. (2019)	Iran	DD	C	L	*S. haemolyticus*	32	ND	ND
Wangai et al. (2019)	Kenya	DD	C	L	*S. haemolyticus*	29	ND	ND
Lee et al. (2019)	ND	MM	C	L	*S. haemolyticus*	31	ND	ND
Sutter et al. (2016)	United States	DD	C	L	*S. haemolyticus*	24,213	ND	ND
Luo et al. (2020)	China	AM	C	L	*S. haemolyticus*	67	ND	25
Tang et al. (2020)	ND	MIC	C	L	*S. haemolyticus*	21	ND	ND
Suneel Kumar et al. (2021)	India	DD	C	L	*S. haemolyticus*	9	ND	ND
Rahimi (2016)	Iran	DD	C	L	*S. haemolyticus*	87	ND	ND
Mehreen et al. (2018)	Pakistan	DD	C	L	*S. haemolyticus*	49	ND	ND
McHardy et al. (2017)	United States	MM	C	L	*S. haemolyticus*	193	ND	ND
Asaad et al. (2016)	ND	AM	C	L	*S. haemolyticus*	23	ND	ND
Javidnia et al. (2015)	Iran	DD	C	L	*S. haemolyticus*	16	ND	ND
Rampelotto et al. (2022)	Brazil	MM	C	L	*S. haemolyticus*	167	ND	ND
Choi et al. (2019)	South Korea	AM	C	L	*S. haemolyticus*	5	ND	ND
Li et al. (2018)	China	MIC	C	L	*S. haemolyticus*	216	ND	ND
Bai et al. (2019)	China	AM	C	L	*S. haemolyticus*	134	ND	ND
Aguinagalde et al. (2015)	India	MIC	E	L	*S. haemolyticus*	ND	190	199
Diriba et al. (2020)	Ethiopia	DD	C	L	*S. haemolyticus*	30	9	ND
Shidiki et al. (2018)	Egypt	DD	ND	H	*S. haemolyticus*	100	ND	ND
Selim et al. (2022)	Saudi Arabia	DD	ND	L	*S. haemolyticus*	100	ND	ND
Sultan et al. (2015)	India	DD	C	L	*S. haemolyticus*	32	ND	ND
Manandhar et al. (2021)	Nepal	DD	C	L	*S. haemolyticus*	127	ND	ND
Mahfouz et al. (2023)	Egypt	DD	C	L	*S. haemolyticus*	52	51	52
Yang et al. (2017)	China	AM	ND	S	*S. haemolyticus*	12	12	12
Soroush et al. (2016)	Iran	DD	C	S	*S. haemolyticus*	68	ND	ND
Hailegiyorgis et al. (2018)	Ethiopia	DD	C	L	*S. haemolyticus*	4	ND	ND
Mesbah Elkammoshi et al. (2016)	Malaysia	DD	C	L	*S. haemolyticus*	179	ND	ND
Agarwal et al. (2016)	India	DD	C	L	*S. haemolyticus*	10	ND	ND
Mama et al. (2019)	Ethiopia	DD	C	L	*S. haemolyticus*	22	ND	ND
Gungor et al. (2021)	Turkey	MIC	E	L	*S. haemolyticus*	36	ND	ND
Ramakrishna et al. (2021)	India	DD	C	L	*S. haemolyticus*	171	ND	ND
Wang et al. (2017)	China	AM	C	L	*S. haemolyticus*	4	ND	ND
Salarvand et al. (2023)	Iran	DD	ND	L	*S. haemolyticus*	88	ND	ND
Firoozeh et al. (2020)	Iran	DD	C	L	*S. haemolyticus*	17	ND	ND
Liu et al. (2015)	China	MIC	C	L	*S. haemolyticus*	116	ND	ND
Fu et al. (2020)	China	AM	C	L	*S. haemolyticus*	189	ND	ND
Akpaka et al. (2017)	Germany	DD	C	L	*S. haemolyticus*	124	ND	ND
Svent-Kucina et al. (2016)	Slovenia	DD	C	L	*S. haemolyticus*	8	ND	ND
Goudarzi et al. (2020)	Iran	DD	C	L	*S. haemolyticus*	86	ND	ND
Fasihi et al. (2016)	Iran	DD	C	L	*S. haemolyticus*	94	ND	ND
Okuda et al. (2016)	Gabon	MIC	E	L	*S. haemolyticus*	8	ND	ND
Ahangarzadeh Rezaee et al. (2016)	Iran	ND	ND	L	*S. haemolyticus*	104	ND	ND
Biset et al. (2020)	Ethiopia	DD	C	L	*S. haemolyticus*	2	ND	ND
Olufunmiso et al. (2017)	Nigeria	DD	C	L	*S. haemolyticus*	122	ND	ND
Tahbaz et al. (2019)	Iran	DD	C	L	*S. haemolyticus*	19	ND	ND
Rukan et al. (2021)	Pakistan	DD	C	L	*S. haemolyticus*	68	ND	ND
Eibach et al. (2017)	Ghana	DD	E	S	*S. haemolyticus*	14	ND	ND
Dayie et al. (2021)	Ghana	DD	C	L	*S. haemolyticus*	5	ND	ND
Salah et al. (2021)	Yemen	AM	ND	L	*S. haemolyticus*	4	ND	ND
Weldu et al. (2020)	Ethiopia	DD	ND	L	*S. haemolyticus*	7	ND	ND
Wan et al. (2016)	Taiwan	MIC	C	L	*S. haemolyticus*	274	ND	ND
John et al. (2023)	Nigeria	DD	C	L	*S. haemolyticus*	6	6	ND
Duncan et al. (2016)	United States	ND	E	L	*S. haemolyticus*	548	ND	ND
Saini et al. (2021)	India	DD	C	L	*S. haemolyticus*	14	ND	ND
Sanchez et al. (2020)	Spain	ND	C	L	*S. haemolyticus*	81	ND	ND
Chen P. F. et al. (2021)	China	MIC	C	L	*S. haemolyticus*	27	ND	ND
Almohammady et al. (2020)	Egypt	DD	C	L	*S. haemolyticus*	15	ND	ND
Iliya et al. (2020)	Kenya	DD	C	L	*S. haemolyticus*	26	ND	ND
Abouelnour et al. (2019)	Egypt	DD	C	S	*S. haemolyticus*	107	ND	ND
Boncompain et al. (2023)	Argentina	DD	C	L	*S. haemolyticus*	7	ND	ND
Al-Tamimi et al. (2021)	Jordan	DD	C	L	*S. haemolyticus*	57	ND	ND
Ullah et al. (2022)	Pakistan	DD	C	S	*S. haemolyticus*	5	ND	ND
Khan et al. (2015)	Nepal	DD	C	L	*S. haemolyticus*	4	ND	ND
Shivappa et al. (2018)	Turkey	DD	C	L	*S. haemolyticus*	7	ND	ND
Muhammad et al. (2020)	Pakistan	DD	C	L	*S. haemolyticus*	ND	ND	14
Kahsay et al. (2018)	Ethiopia	DD	C	L	*S. haemolyticus*	6	ND	ND
Liang et al. (2018)	China	AM	C	L	*S. haemolyticus*	26	ND	ND
Zhang et al. (2015)	China	DD	C	L	*S. haemolyticus*	58	ND	ND
El-Kersh et al. (2016)	Saudi Arabia	AM	C	L	*S. haemolyticus*	5	ND	ND
Fateh Dizji et al. (2023)	Iran	DD	C	L	*S. haemolyticus*	45	ND	ND
Baz et al. (2021)	Egypt	DD	ND	L	*S. haemolyticus*	39	38	ND
Vijay and Dalela (2016)	India	DD	C	L	*S. haemolyticus*	14	ND	ND
AL-Salihi et al. (2023)	Iraq	DD	C	L	*S. haemolyticus*	6	ND	ND
Joachim et al. (2017)	Tanzania	DD	C	L	*S. haemolyticus*	11	ND	ND
Goes et al. (2021)	Brazil	DD	C	L	*S. haemolyticus*	29	ND	ND
Sapkota et al. (2019)	Nepal	DD	C	L	*S. haemolyticus*	16	ND	ND
Abdulmanea et al. (2023)	Saudi Arabia	AM	C	L	*S. haemolyticus*	30	ND	9
Adhikari et al. (2023)	Nepal	DD	C	L	*S. haemolyticus*	226	ND	ND
Zhou et al. (2020)	China	AM	C	L	*S. haemolyticus*	17	ND	ND
Kim et al. (2020)	South Korea	DD	C	L	*S. haemolyticus*	ND	ND	14
El-Amir et al. (2019)	Egypt	DD	C	L	*S. haemolyticus*	ND	ND	2
Arabestani et al. (2018)	Iran	DD	C	L	*S. haemolyticus*	160	ND	ND
Roden et al. (2019)	ND	ND	ND	L	*S. haemolyticus*	5	ND	ND
Al-Humaidan et al. (2015)	Saudi Arabia	DD	C	L	*S. haemolyticus*	5	ND	ND
Mansson et al. (2015)	Sweden	DD	E	L	*S. haemolyticus*	6	ND	ND
Garza-Gonzalez et al. (2019)	Mexico	DD	C	L	*S. haemolyticus*	871	ND	ND
Bhatt et al. (2016)	China	DD	C	L	*S. haemolyticus*	81	ND	ND
Maina et al. (2016)	Kenya	AM	C	L	*S. haemolyticus*	36	ND	ND
Wurster et al. (2018)	United States	ND	C	L	*S. haemolyticus*	107	ND	ND
Cavalcante et al. (2020)	Brazil	ND	ND	H	*S. haemolyticus*	9	ND	ND
Taha et al. (2019)	Sweden	DD	E	L	*S. haemolyticus*	506	ND	ND
Kurup and Ansari (2019)	Guyana	DD	C	L	*S. haemolyticus*	14	ND	ND
Kulshrestha et al. (2021)	India	DD	C	S	*S. haemolyticus*	25	ND	ND
Mottola et al. (2016)	Portugal	MIC	C	L	*S. haemolyticus*	8	ND	ND
Lenart-Boron et al. (2016)	Poland	DD	E	L	*S. haemolyticus*	23	ND	ND
Uyar Güleç et al. (2020)	Turkey	MIC	C	L	*S. haemolyticus*	45	ND	ND
Al-Qaisi and Al-Salmani (2020)	Iraq	DD	C	S	*S. haemolyticus*	50	ND	ND
Kpeli et al. (2016)	Ghana	ND	C	L	*S. haemolyticus*	15	ND	ND
Demir et al. (2020)	Turkey	DD	E	L	*S. haemolyticus*	30	ND	ND
Singh and Hota (2019)	India	AM	C	L	*S. haemolyticus*	8	ND	ND
Dilnessa and Bitew (2016)	Ethiopia	DD	C	L	*S. haemolyticus*	3	ND	ND
Rajkumar et al. (2017)	India	DD	C	L	*S. haemolyticus*	3,058	ND	ND
Hoffmann et al. (2015)	Austria	MM	E	L	*S. haemolyticus*	73	ND	74
Kumar and Shetty (2021)	India	DD	C	L	*S. haemolyticus*	43	ND	ND
Ahmad et al. (2020)	India	DD	C	L	*S. haemolyticus*	ND	ND	3
Juda et al. (2016)	Poland	DD	E	L	*S. haemolyticus*	75	ND	ND
Ibadin et al. (2017)	ND	DD	C	L	*S. haemolyticus*	48	ND	ND
Tsige et al. (2020)	Ethiopia	DD	ND	L	*S. haemolyticus*	25	ND	ND
Banawas et al. (2023)	Saudi Arabia	MM	ND	L	*S. haemolyticus*	27	ND	ND
Mascaro et al. (2019)	Italy	DD	E	L	*S. aureus*	16	ND	ND
Lennartz et al. (2019)	Germany	DD	E	L	*S. aureus*	42	ND	ND
Al Zebary et al. (2017)	Iraq	DD	NCCLS	L	*S. aureus*	10	ND	ND
Sakabe and Del Fiol Fde (2016)	Brazil	ND	ND	L	*S. aureus*	5	ND	ND
Lin et al. (2018)	China	DD	C	L	*S. aureus*	28	ND	ND
Doss et al. (2017)	Egypt	DD	C	L	*S. aureus*	13	7	10
Liang et al. (2023)	China	AM	C	L	MSSA	127	ND	ND
Oydanich et al. (2017)	United States	AM	C	L	*S. aureus*	62	ND	ND
Gajdacs et al. (2021)	Hungary	ND	E	S	*Staphylococcus Spp*	ND	ND	67
Mostafa et al. (2015)	Iran	DD	C	L	*S. aureus*	95	ND	69
Soumya et al. (2017)	India	DD	ND	S	*S. epidermidis*	152	ND	ND
Parastan et al. (2020)	Iran	DD	C	L	*S. aureus*	93	ND	ND
Farah et al. (2019)	Saudi Arabia	MIC	C	L	*S. aureus*	507	ND	ND
Sotoudeh Anvari et al. (2015)	Iran	DD	C	L	*S. epidermidis*	13	ND	ND

AST method (Multiple Method, MM), Disk Diffusion (DD), Minimum Inhibitory Concentration (MIC) method, and Automated Method (AM). Publication Bias: Risk (S), Low Risk (L), High Risk (H). AST guideline: CLSI: C, EUCAST: E.

### 3.2 Comprehensive overview of antibiotic resistance prevalence

Among 360 reports, the proportion of erythromycin-resistant isolates was 0.573 (95% CI: 0.556–0.590), based on 144,746 resistant isolates out of 293,411 isolates tested. The heterogeneity among reports was significant (I^2^ = 96.09%, *p* = 0.001). Similarly, the proportion of clarithromycin resistance, as assessed by 30 reports involving 4,015 resistant isolates out of 8,045 tested isolates, was 0.526 (95% CI: 0.380–0.668), with significant heterogeneity between reports (I^2^ = 98.76%, *p* = 0.001). In addition, the proportion of azithromycin-resistant isolates, derived from 83 reports containing 5,227 resistant isolates out of 10,553 isolates tested, was 0.579 (95% CI: 0.514–0.641), again with significant heterogeneity between reports (I^2^ = 96.50%, *p* = 0.001).

#### 3.2.1 Prevalence of erythromycin resistance

**Figure 2:**
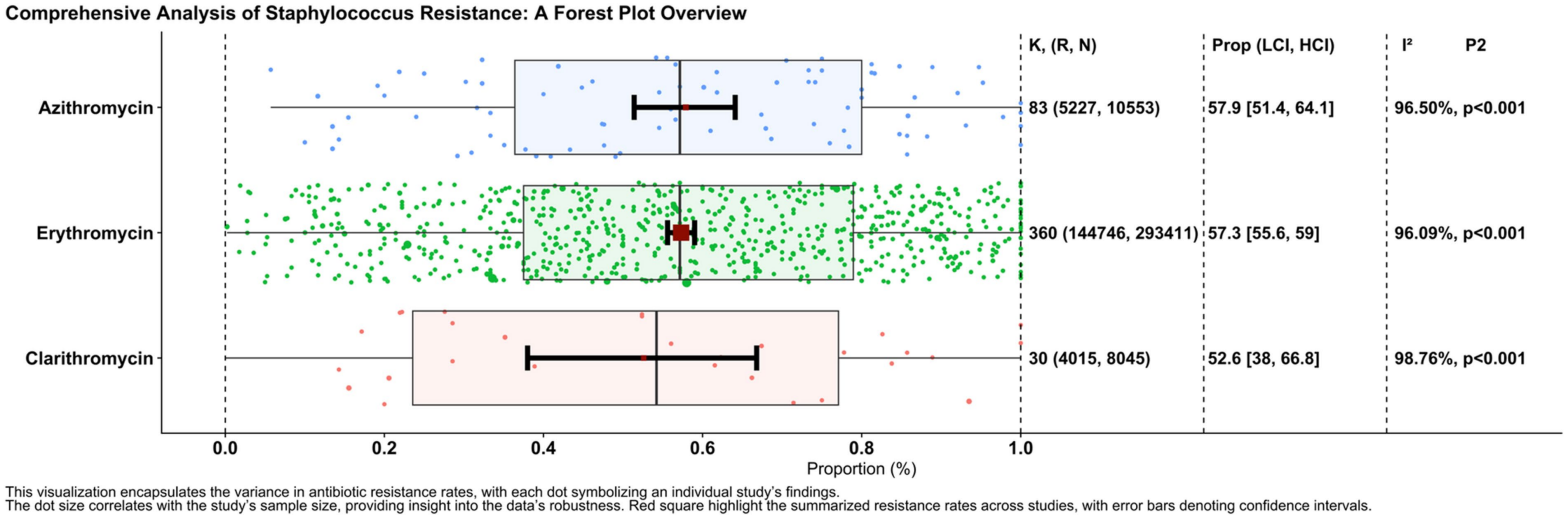
Forest plot of resistance rates for macrolide antibiotics against *Staphylococcus*: the forest plot summarizes the resistance rates of *Staphylococcus* species to Azithromycin, Erythromycin, and Clarithromycin across various studies. Each dot represents an individual study’s data point, with red squares indicating pooled resistance estimates and black bars showing confidence intervals.

A total of 293,411 isolates from 721 studies were included in the erythromycin resistance analysis. The estimated mean proportion based on the random effects model was 0.573 (95% CI: 0.556–0.590). This result indicates that the mean proportion differed significantly from zero (z = 8.400, *p* < 0.001). The heterogeneity between studies was significant, as noted in the Q-test (Q(720) = 42,007.095, I^2^ = 98.29%, *p* < 0.001) (Table 2). A forest plot illustrating the observed results and the random effects model estimate is shown in Figure 2. Using the trim-and-fill method, the adjusted proportion was 0.501 (95% CI: 0.483–0.518). Analysis of the studentized residuals identified several studies with values greater than 3.979, suggesting potential outliers within the model. After excluding these potential outliers, the proportion was 0.501 (95% CI: 0.483–0.518). Cook’s distance analysis also indicated that several studies were overly influential. After removing these influential studies, the proportion remained unchanged at 0.501 (95% CI: 0.483–0.518). Both the rank correlation test and the regression test suggested a potential funnel plot asymmetry (*p* < 0.001 and *p* = 0.018, respectively) (Table 3).

**Table 2:** Meta-analysis statistics of worldwide antibiotic resistance in *Staphylococcus* spp.

Antibiotic	K (n, N)	Proportion 95%CI (LCI, HCI)	I^2^	P1	P2
Erythromycin	721 (144,746, 293,411)	0.573 (0.556, 0.590)	98.29%	*p* < 0.001	*p* < 0.001
Clarithromycin	30 (4,015, 8,045)	0.526 (0.380, 0.668)	98.76%	*p* = 0.727	*p* < 0.001
Azithromycin	83 (5,227, 10,553)	0.579 (0.514, 0.641)	96.50%	*p* = 0.017	*p* < 0.001

K: Number of reports, n: Number of resistant isolates, N: Number of total isolates, LCI: 95% Lower Limit Confidence Interval, HCI: 95% Higher Limit Confidence Interval, P1: *p*-value of difference from zero resistance rate, P2: *p*-value of heterogeneity between reports.

**Table 3:** Evaluation of publication bias in meta-analysis.

Antibiotic	Egger test	Begg test	Fail and safe	Trim and Fill
Erythromycin	*p* < 0.001	*p* = 0.837	104,799	0.501 (0.483, 0.518)
Clarithromycin	*p* = 0.890	*p* = 0.432	0	0.526 (0.380, 0.668)
Azithromycin	*p* < 0.001	*p* = 0.264	473	0.519 (0.455, 0.582)

This table provides a comprehensive assessment of potential publication bias in the meta-analysis using a range of statistical techniques. Included are statistics generated from Egger’s Method, Begg’s Method, the Fail-Safe N (NFS), and the Trim-and-Fill Method. These methods are applied to investigate the presence of bias and its impact on the meta-analysis results, ensuring the robustness and reliability of the findings.

#### 3.2.2 Prevalence of clarithromycin resistance

**Figure 3:**
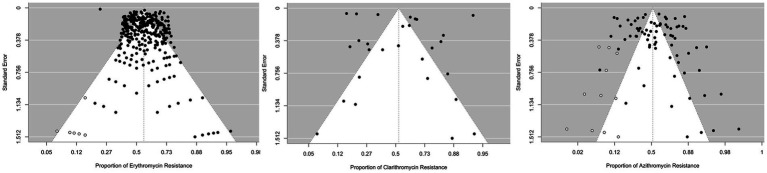
Funnel plots for publication bias analysis: funnel plots assessing the presence of publication bias in resistance studies for Erythromycin (left), Clarithromycin (middle), and Azithromycin (right). Symmetrical distributions indicate minimal bias, whereas asymmetries may suggest potential bias.

The clarithromycin resistance analysis included Eight forty-five isolates from 30 studies. The estimated average proportion based on the random-effects model was 0.526 (95%CI, 0.380, 0.668). Therefore, the average outcome was not significantly different from zero (z = 0.349, *p* = 0.727). According to the Q test, the outcomes were heterogeneous (Q (29) = 2347.241, I ^2^ = 98.76%, *p* < 0.001). A forest plot showing the observed outcomes and the estimate based on the random effects model is shown in Figure 2. With the fill and trim method implementation, the proportion changed to 0.526 (95%CI, 0.380, 0.668). Examination of the studentized residuals revealed that none of the studies had values greater than 3.144. Hence, there was no indication of outliers in the context of this model. According to Cook’s distance, none of the studies could be considered overly influential. Neither the rank correlation nor the regression test indicated funnel plot asymmetry (*p* = 0.432 and *p* = 0.890, respectively) (Figure 3).

#### 3.2.3 Prevalence of azithromycin resistance

The analysis of azithromycin resistance included data from 83 studies with 10,553 isolates. Using a random effects model, the estimated mean proportion was 0.579 (95% CI: 0.514, 0.641), indicating that the mean outcome differed significantly from zero (*z* = 2.385, *p* = 0.017). The heterogeneity of the outcomes was confirmed by the Q-test (Q(82) = 2342.061, I^2^ = 96.50%, *p* < 0.001). After using the fill-and-trim method, the proportion was adjusted to 0.519 (95% CI: 0.455, 0.582). Analysis of the studentized residuals showed no study exceeded a value of 3.431, indicating no outliers in the model. Furthermore, Cook’s distance analysis indicated that no single study had an undue influence on the results. While the regression test revealed funnel plot asymmetry (*p* < 0.001), the rank correlation test did not reveal significant asymmetry (*p* = 0.264).

### 3.3 Subgroup analysis

This section provides a detailed summary of the subgroup analyses performed on antimicrobial resistance. The full dataset is available in Table 4. The analyses examined variations in resistance rates across geographic regions, antimicrobial susceptibility testing (AST) methods, time trends, and study quality.

**Table 4:** Meta-analysis statistics of worldwide antibiotic resistance in staphylococcus spp. and subgroup analysis results.

Category	Subgroup	K (n, N)	Proportion 95%CI (LCI, HCI)	I^2^	P1	P2	P3
Erythromycin							
Overall	ND	360 (144,746, 293,411)	0.573 (0.556, 0.590)	96.09%	*p* < 0.001	*p* < 0.001	NA
Countries	China	105 (11,791, 18,008)	0.731 (0.692, 0.766)	96.02%	*p* < 0.001	*p* < 0.001	p < 0.001
	Nepal	24 (3,996, 6,515)	0.620 (0.568, 0.669)	92.18%	*p* < 0.001	*p* < 0.001	
	Rwanda	1 (25, 138)	0.181 (0.125, 0.254)	0.00%	*p* < 0.001	*p* > 0.999	
	Iran	85 (5,568, 9,107)	0.627 (0.579, 0.671)	93.57%	*p* < 0.001	*p* < 0.001	
	Kuwait	4 (4,805, 11,978)	0.421 (0.370, 0.474)	94.75%	*p* = 0.004	*p* < 0.001	
	Ethiopia	35 (582, 1,493)	0.431 (0.345, 0.522)	86.72%	*p* = 0.137	*p* < 0.001	
	India	79 (7,667, 14,709)	0.557 (0.514, 0.599)	94.19%	*p* = 0.010	*p* < 0.001	
	Cameroon	1 (111, 201)	0.552 (0.483, 0.620)	0.00%	*p* = 0.139	*p* > 0.999	
	South Korea	11 (1,155, 1786)	0.576 (0.333, 0.787)	97.89%	*p* = 0.550	*p* < 0.001	
	Poland	8 (333, 873)	0.350 (0.235, 0.486)	91.39%	*p* = 0.031	*p* < 0.001	
	Spain	13 (12,781, 39,342)	0.425 (0.304, 0.556)	98.27%	*p* = 0.262	*p* < 0.001	
	Malaysia	8 (1,587, 2070)	0.704 (0.421, 0.886)	98.64%	*p* = 0.151	*p* < 0.001	
	Kenya	8 (475, 766)	0.517 (0.323, 0.705)	94.92%	*p* = 0.872	*p* < 0.001	
	United States	28 (48,494, 84,187)	0.558 (0.495, 0.618)	99.50%	*p* = 0.070	*p* < 0.001	
	Australia	2 (199, 477)	0.720 (0.121, 0.979)	93.84%	*p* = 0.527	*p* < 0.001	
	Hungary	1 (122, 153)	0.797 (0.726, 0.854)	0.00%	*p* < 0.001	*p* > 0.999	
	Nigeria	23 (857, 1,353)	0.626 (0.523, 0.718)	89.63%	*p* = 0.017	*p* < 0.001	
	Taiwan	7 (2,849, 5,223)	0.724 (0.331, 0.933)	99.71%	*p* = 0.257	*p* < 0.001	
	Colombia	2 (144, 353)	0.260 (0.055, 0.681)	94.04%	*p* = 0.255	*p* < 0.001	
	Libya	1 (30, 32)	0.938 (0.782, 0.984)	0.00%	*p* < 0.001	*p* > 0.999	
	Switzerland	4 (127, 243)	0.536 (0.457, 0.613)	21.30%	*p* = 0.377	*p* = 0.283	
	Pakistan	9 (470, 762)	0.703 (0.482, 0.857)	95.53%	*p* = 0.070	*p* < 0.001	
	Eritrea	2 (14, 102)	0.159 (0.067, 0.331)	60.43%	*p* < 0.001	*p* = 0.112	
	Oman	2 (12, 60)	0.194 (0.089, 0.371)	32.07%	*p* = 0.002	*p* = 0.225	
	Croatia	1 (523, 542)	0.965 (0.946, 0.978)	0.00%	*p* < 0.001	*p* > 0.999	
	Brazil	17 (1,013, 1740)	0.546 (0.436, 0.651)	93.59%	*p* = 0.412	*p* < 0.001	
	Ghana	11 (133, 833)	0.165 (0.117, 0.226)	74.51%	*p* < 0.001	*p* < 0.001	
	Denmark	2 (185, 1856)	0.182 (0.030, 0.614)	99.08%	*p* = 0.134	*p* < 0.001	
	Japan	2 (216, 223)	0.966 (0.931, 0.983)	0.00%	*p* < 0.001	*p* = 0.431	
	Philippines	1 (3, 108)	0.028 (0.009, 0.083)	0.00%	*p* < 0.001	*p* > 0.999	
	Thailand	2 (39, 43)	0.900 (0.761, 0.962)	0.00%	*p* < 0.001	*p* = 0.431	
	Palestinian Territories	5 (539, 870)	0.569 (0.452, 0.680)	90.18%	*p* = 0.248	*p* < 0.001	
	Turkey	10 (1,371, 2,736)	0.626 (0.464, 0.765)	94.97%	*p* = 0.127	*p* < 0.001	
	Canada	1 (521, 535)	0.974 (0.956, 0.984)	0.00%	*p* < 0.001	*p* > 0.999	
	Israel	2 (274, 451)	0.358 (0.031, 0.906)	98.19%	*p* = 0.687	*p* < 0.001	
	Jordan	5 (124, 170)	0.787 (0.450, 0.943)	89.75%	*p* = 0.089	*p* < 0.001	
	Egypt	16 (987, 1,409)	0.788 (0.689, 0.863)	92.58%	*p* < 0.001	*p* < 0.001	
	Iraq	23 (749, 1,445)	0.565 (0.470, 0.656)	88.97%	*p* = 0.177	*p* < 0.001	
	Saudi Arabia	19 (1,141, 4,320)	0.610 (0.410, 0.778)	97.77%	*p* = 0.279	*p* < 0.001	
	Portugal	7 (295, 590)	0.535 (0.398, 0.666)	87.32%	*p* = 0.622	*p* < 0.001	
	Serbia	1 (27, 50)	0.540 (0.402, 0.672)	0.00%	*p* = 0.572	*p* > 0.999	
	Algeria	2 (18, 72)	0.250 (0.164, 0.362)	0.00%	*p* < 0.001	*p* > 0.999	
	South Africa	5 (208, 400)	0.530 (0.317, 0.733)	90.36%	*p* = 0.788	*p* < 0.001	
	Argentina	5 (86, 181)	0.479 (0.355, 0.605)	59.85%	*p* = 0.742	*p* = 0.041	
	Guyana	2 (52, 72)	0.737 (0.213, 0.967)	92.79%	*p* = 0.388	*p* < 0.001	
	Mexico	7 (1,448, 4,153)	0.522 (0.408, 0.634)	95.37%	*p* = 0.702	*p* < 0.001	
	France	2 (106, 227)	0.270 (0.037, 0.780)	79.93%	*p* = 0.389	*p* = 0.026	
	Qatar	1 (19, 20)	0.950 (0.718, 0.993)	0.00%	*p* = 0.004	*p* > 0.999	
	Russia	1 (19, 27)	0.704 (0.510, 0.844)	0.00%	*p* = 0.040	*p* > 0.999	
	Vietnam	5 (313, 408)	0.763 (0.619, 0.864)	85.59%	*p* < 0.001	*p* < 0.001	
	Afghanistan	1 (11, 98)	0.112 (0.063, 0.191)	0.00%	*p* < 0.001	*p* > 0.999	
	Uganda	4 (182, 303)	0.621 (0.355, 0.830)	88.22%	*p* = 0.375	*p* < 0.001	
	United Arab Emirates	1 (1, 3)	0.333 (0.043, 0.846)	0.00%	*p* = 0.571	*p* > 0.999	
	Italy	7 (664, 1,434)	0.408 (0.301, 0.525)	93.28%	*p* = 0.124	*p* < 0.001	
	Burkina Faso	1 (21, 149)	0.141 (0.094, 0.207)	0.00%	*p* < 0.001	*p* > 0.999	
	Mozambique	1 (84, 236)	0.356 (0.297, 0.419)	0.00%	*p* < 0.001	*p* > 0.999	
	Romania	1 (835, 1,672)	0.499 (0.475, 0.523)	0.00%	*p* = 0.961	*p* > 0.999	
	Norway	2 (58, 375)	0.173 (0.068, 0.373)	92.52%	*p* = 0.003	*p* < 0.001	
	Indonesia	2 (139, 211)	0.645 (0.541, 0.738)	27.49%	*p* = 0.007	*p* = 0.240	
	Kazakhstan	1 (1, 5)	0.200 (0.027, 0.691)	0.00%	*p* = 0.215	*p* > 0.999	
	Tanzania	3 (70, 249)	0.280 (0.133, 0.495)	87.51%	*p* = 0.045	*p* < 0.001	
	United Kingdom	2 (203, 631)	0.392 (0.219, 0.596)	77.49%	*p* = 0.298	*p* = 0.035	
	Tunisia	2 (21, 99)	0.215 (0.028, 0.722)	93.70%	*p* = 0.258	*p* < 0.001	
	Uruguay	1 (5, 100)	0.050 (0.021, 0.115)	0.00%	*p* < 0.001	*p* > 0.999	
	Germany	5 (394, 1,695)	0.283 (0.181, 0.413)	94.22%	*p* = 0.002	*p* < 0.001	
	Slovenia	1 (8, 274)	0.029 (0.015, 0.057)	0.00%	*p* < 0.001	*p* > 0.999	
	Gabon	1 (8, 103)	0.078 (0.039, 0.148)	0.00%	*p* < 0.001	*p* > 0.999	
	Greece	2 (343, 715)	0.398 (0.217, 0.612)	88.85%	*p* = 0.350	*p* = 0.003	
	Yemen	1 (4, 11)	0.364 (0.143, 0.661)	0.00%	*p* = 0.372	*p* > 0.999	
	Austria	2 (146, 1,098)	0.133 (0.114, 0.154)	0.00%	*p* < 0.001	*p* > 0.999	
	Gambia	1 (26, 293)	0.089 (0.061, 0.127)	0.00%	*p* < 0.001	*p* > 0.999	
	Bangladesh	1 (19, 29)	0.655 (0.469, 0.803)	0.00%	*p* = 0.100	*p* > 0.999	
	Niger	1 (4, 10)	0.400 (0.158, 0.703)	0.00%	*p* = 0.530	*p* > 0.999	
	Bulgaria	2 (296, 870)	0.340 (0.309, 0.372)	0.00%	*p* < 0.001	*p* > 0.999	
	Sweden	2 (512, 572)	0.654 (0.031, 0.991)	98.64%	*p* = 0.759	*p* < 0.001	
	Myanmar (Burma)	1 (86, 153)	0.562 (0.483, 0.639)	0.00%	*p* = 0.126	*p* > 0.999	
Continents	Asia	417 (44,949, 81,522)	0.638 (0.616, 0.660)	96.87%	*p* < 0.001	*p* < 0.001	p < 0.001
	Africa	119 (3,856, 8,241)	0.476 (0.423, 0.529)	93.22%	*p* = 0.373	*p* < 0.001	
	ND	54 (26,002, 58,611)	0.535 (0.453, 0.616)	99.54%	*p* = 0.399	*p* < 0.001	
	Europe	66 (17,977, 53,239)	0.407 (0.359, 0.456)	97.82%	*p* < 0.001	*p* < 0.001	
	Americas	63 (51,763, 91,321)	0.544 (0.499, 0.588)	99.08%	*p* = 0.057	*p* < 0.001	
	Oceania	2 (199, 477)	0.720 (0.121, 0.979)	93.84%	*p* = 0.527	*p* < 0.001	
AST Guideline	CLSI	563 (114,948, 218,991)	0.584 (0.565, 0.604)	98.25%	*p* < 0.001	*p* < 0.001	p < 0.001
	EUCAST	67 (24,762, 66,311)	0.430 (0.382, 0.480)	98.71%	*p* = 0.006	*p* < 0.001	
	Multiple Guideline	8 (778, 1,415)	0.507 (0.314, 0.697)	97.26%	*p* = 0.946	*p* < 0.001	
	NCCLS	6 (398, 871)	0.353 (0.200, 0.543)	90.72%	*p* = 0.128	*p* < 0.001	
	ND	74 (3,686, 5,519)	0.660 (0.601, 0.715)	92.23%	*p* < 0.001	*p* < 0.001	
	BSAC	1 (65, 79)	0.823 (0.723, 0.892)	0.00%	*p* < 0.001	*p* > 0.999	
	FMS	1 (4, 10)	0.400 (0.158, 0.703)	0.00%	*p* = 0.530	*p* > 0.999	
	CASFM	1 (105, 215)	0.488 (0.422, 0.555)	0.00%	*p* = 0.733	*p* > 0.999	
AST method	Automate	98 (9,062, 14,658)	0.660 (0.612, 0.705)	95.75%	*p* < 0.001	*p* < 0.001	p = 0.001
	Disk Diffusion	452 (72,252, 128,319)	0.557 (0.537, 0.576)	96.69%	*p* < 0.001	*p* < 0.001	
	MIX	73 (34,775, 77,633)	0.566 (0.507, 0.624)	99.44%	*p* = 0.028	*p* < 0.001	
	MIC	59 (25,047, 65,498)	0.568 (0.510, 0.624)	98.98%	*p* = 0.023	*p* < 0.001	
Species	MRSA	212 (41,180, 58,142)	0.710 (0.679, 0.740)	97.67%	*p* < 0.001	*p* < 0.001	p < 0.001
	*S. saprophyticus*	2 (91, 181)	0.593 (0.320, 0.819)	74.47%	*p* = 0.514	*p* = 0.048	
	*Staphylococcus spp*	19 (955, 1997)	0.522 (0.423, 0.619)	92.87%	*p* = 0.662	*p* < 0.001	
	*S. hominis*	5 (125, 166)	0.751 (0.678, 0.812)	0.00%	*p* < 0.001	*p* = 0.448	
	CoNS	42 (4,066, 7,352)	0.568 (0.505, 0.629)	95.30%	*p* = 0.034	*p* < 0.001	
	*S. lugdunensis*	4 (236, 1,142)	0.313 (0.144, 0.552)	91.74%	*p* = 0.121	*p* < 0.001	
	*S. aureus*	342 (92,286, 210,496)	0.496 (0.475, 0.516)	98.33%	*p* = 0.680	*p* < 0.001	
	*S. haemolyticus*	8 (500, 692)	0.787 (0.544, 0.919)	94.55%	*p* = 0.023	*p* < 0.001	
	*S. epidermidis*	41 (1953, 2,818)	0.676 (0.601, 0.744)	90.89%	*p* < 0.001	*p* < 0.001	
	MSSA	37 (2,868, 9,758)	0.305 (0.221, 0.404)	98.29%	*p* < 0.001	*p* < 0.001	
	MRCoNS	5 (381, 488)	0.777 (0.526, 0.916)	94.78%	*p* = 0.032	*p* < 0.001	
	MSCoNS	1 (10, 69)	0.145 (0.080, 0.249)	0.00%	*p* < 0.001	*p* > 0.999	
	VSSA	1 (57, 61)	0.934 (0.838, 0.975)	0.00%	*p* < 0.001	*p* > 0.999	
	VISA	1 (11, 11)	0.958 (0.575, 0.997)	0.00%	*p* = 0.030	*p* > 0.999	
	*S. capitis*	1 (27, 38)	0.711 (0.549, 0.832)	0.00%	*p* = 0.012	*p* > 0.999	
Coagulase	CPS	593 (136,402, 278,468)	0.565 (0.546, 0.584)	98.49%	*p* < 0.001	*p* < 0.001	*p* = 0.021
	CoNS	109 (7,389, 12,946)	0.632 (0.584, 0.677)	95.26%	*p* < 0.001	*p* < 0.001	
	ND	19 (955, 1997)	0.522 (0.423, 0.619)	92.87%	*p* = 0.662	*p* < 0.001	
year group	2020_2023	379 (62,408, 148,526)	0.550 (0.525, 0.575)	98.32%	*p* < 0.001	*p* < 0.001	p = 0.002
	2015_2019	342 (82,338, 144,885)	0.596 (0.575, 0.616)	97.62%	*p* < 0.001	*p* < 0.001	
Clarithromycin							
Overall	ND	30 (4,015, 8,045)	0.526 (0.380, 0.668)	98.76%	*p* = 0.727	*p* < 0.001	NA
Countries	Canada	2 (590, 3,348)	0.179 (0.135, 0.234)	92.92%	*p* < 0.001	*p* < 0.001	p < 0.001
	Japan	4 (2,261, 2,455)	0.660 (0.249, 0.920)	97.28%	*p* = 0.462	*p* < 0.001	
	Egypt	5 (105, 171)	0.590 (0.358, 0.788)	79.21%	*p* = 0.452	*p* < 0.001	
	Iran	3 (28, 77)	0.388 (0.177, 0.651)	78.75%	*p* = 0.407	*p* = 0.009	
	India	5 (896, 1735)	0.612 (0.438, 0.761)	97.37%	*p* = 0.205	*p* < 0.001	
	Kazakhstan	1 (1, 5)	0.200 (0.027, 0.691)	0.00%	*p* = 0.215	*p* > 0.999	
	Nigeria	4 (37, 56)	0.666 (0.407, 0.852)	55.45%	*p* = 0.205	*p* = 0.081	
	Ethiopia	2 (17, 70)	0.244 (0.157, 0.358)	0.00%	*p* < 0.001	*p* = 0.589	
	China	3 (79, 121)	0.729 (0.490, 0.883)	44.92%	*p* = 0.060	*p* = 0.163	
	Pakistan	1 (1, 7)	0.143 (0.020, 0.581)	0.00%	*p* = 0.097	*p* > 0.999	
Continents	Americas	2 (590, 3,348)	0.179 (0.135, 0.234)	92.92%	*p* < 0.001	*p* < 0.001	*p* = 0.095
	Asia	17 (3,266, 4,400)	0.580 (0.404, 0.738)	98.29%	*p* = 0.372	*p* < 0.001	
	Africa	11 (159, 297)	0.529 (0.358, 0.693)	81.76%	*p* = 0.747	*p* < 0.001	
AST Guideline	CLSI	24 (3,467, 7,231)	0.453 (0.291, 0.626)	98.92%	*p* = 0.599	*p* < 0.001	*p* = 0.115
	ND	4 (115, 137)	0.837 (0.765, 0.889)	0.00%	*p* < 0.001	*p* = 0.989	
	EUCAST	2 (433, 677)	0.640 (0.601, 0.677)	8.00%	*p* < 0.001	*p* = 0.297	
AST method	MIX	3 (597, 3,355)	0.192 (0.136, 0.263)	91.13%	*p* < 0.001	*p* < 0.001	p = 0.060
	Disk Diffusion	19 (707, 1,511)	0.503 (0.385, 0.620)	90.51%	*p* = 0.964	*p* < 0.001	
	MIC	6 (2,696, 3,162)	0.614 (0.352, 0.824)	98.76%	*p* = 0.396	*p* < 0.001	
	Automate	2 (15, 17)	0.861 (0.619, 0.959)	0.00%	*p* = 0.007	*p* = 0.927	
Species	MRSA	6 (576, 2,353)	0.607 (0.269, 0.867)	98.70%	*p* = 0.552	*p* < 0.001	*p* = 0.582
	*S. aureus*	12 (2,630, 3,066)	0.632 (0.422, 0.802)	97.35%	*p* = 0.216	*p* < 0.001	
	MSSA	2 (560, 2,167)	0.273 (0.154, 0.436)	98.16%	*p* = 0.008	*p* < 0.001	
	*S. epidermidis*	3 (15, 46)	0.560 (0.113, 0.927)	82.89%	*p* = 0.837	*p* = 0.003	
	CoNS	3 (199, 335)	0.320 (0.074, 0.735)	93.00%	*p* = 0.404	*p* < 0.001	
	*Staphylococcus spp*	3 (29, 57)	0.439 (0.132, 0.802)	85.03%	*p* = 0.772	*p* = 0.001	
	*S. lugdunensis*	1 (6, 21)	0.286 (0.134, 0.508)	0.00%	*p* = 0.058	*p* > 0.999	
Coagulase	CPS	20 (3,766, 7,586)	0.581 (0.398, 0.745)	99.15%	*p* = 0.385	*p* < 0.001	*p* = 0.570
	CoNS	7 (220, 402)	0.392 (0.180, 0.655)	89.36%	*p* = 0.426	*p* < 0.001	
	ND	3 (29, 57)	0.439 (0.132, 0.802)	85.03%	*p* = 0.772	*p* = 0.001	
Year Group	2020_2023	17 (946, 3,990)	0.405 (0.281, 0.543)	96.40%	*p* = 0.177	*p* < 0.001	p = 0.032
	2015_2019	13 (3,069, 4,055)	0.674 (0.467, 0.830)	98.65%	*p* = 0.098	*p* < 0.001	
Azithromycin							
Overall	ND	83 (5,227, 10,553)	0.579 (0.514, 0.641)	96.50%	*p* = 0.017	*p* < 0.001	NA
Countries	United States	6 (630, 1,511)	0.452 (0.296, 0.618)	96.64%	*p* = 0.577	*p* < 0.001	p = 0.009
	Nepal	2 (94, 162)	0.554 (0.402, 0.696)	65.84%	*p* = 0.487	*p* = 0.087	
	Spain	2 (170, 883)	0.348 (0.033, 0.894)	99.34%	*p* = 0.656	*p* < 0.001	
	India	17 (1910, 3,360)	0.575 (0.458, 0.685)	96.84%	*p* = 0.207	*p* < 0.001	
	China	8 (916, 1,137)	0.768 (0.569, 0.893)	94.57%	*p* = 0.011	*p* < 0.001	
	Brazil	2 (65, 108)	0.808 (0.050, 0.997)	94.54%	*p* = 0.520	*p* < 0.001	
	Egypt	6 (92, 149)	0.609 (0.417, 0.773)	57.17%	*p* = 0.262	*p* = 0.040	
	Pakistan	5 (64, 75)	0.831 (0.722, 0.903)	0.00%	*p* < 0.001	*p* = 0.746	
	Bangladesh	6 (88, 182)	0.500 (0.398, 0.601)	41.23%	*p* = 0.993	*p* = 0.130	
	Iran	11 (469, 805)	0.563 (0.475, 0.648)	80.97%	*p* = 0.160	*p* < 0.001	
	Iraq	3 (108, 150)	0.770 (0.306, 0.962)	92.89%	*p* = 0.243	*p* < 0.001	
	Saudi Arabia	2 (45, 93)	0.481 (0.060, 0.930)	96.42%	*p* = 0.954	*p* < 0.001	
	Malaysia	1 (61, 209)	0.292 (0.234, 0.357)	0.00%	*p* < 0.001	*p* > 0.999	
	Kazakhstan	1 (1, 5)	0.200 (0.027, 0.691)	0.00%	*p* = 0.215	*p* > 0.999	
	Indonesia	1 (12, 22)	0.545 (0.341, 0.735)	0.00%	*p* = 0.670	*p* > 0.999	
	South Africa	1 (66, 89)	0.742 (0.641, 0.822)	0.00%	*p* < 0.001	*p* > 0.999	
	Austria	2 (148, 1,098)	0.135 (0.116, 0.156)	0.00%	*p* < 0.001	*p* > 0.999	
	South Korea	1 (14, 25)	0.560 (0.366, 0.737)	0.00%	*p* = 0.549	*p* > 0.999	
	Australia	1 (58, 63)	0.921 (0.823, 0.967)	0.00%	*p* < 0.001	*p* > 0.999	
	Hungary	2 (94, 172)	0.533 (0.209, 0.830)	95.14%	*p* = 0.861	*p* < 0.001	
Continents	Americas	8 (695, 1,619)	0.493 (0.345, 0.643)	95.81%	*p* = 0.929	*p* < 0.001	*p* = 0.013
	Asia	58 (3,782, 6,225)	0.604 (0.540, 0.666)	94.36%	*p* = 0.002	*p* < 0.001	
	Europe	6 (412, 2,153)	0.311 (0.149, 0.537)	98.29%	*p* = 0.098	*p* < 0.001	
	ND	3 (122, 255)	0.466 (0.053, 0.932)	97.70%	*p* = 0.923	*p* < 0.001	
	Africa	7 (158, 238)	0.641 (0.485, 0.772)	66.45%	*p* = 0.076	*p* = 0.007	
	Oceania	1 (58, 63)	0.921 (0.823, 0.967)	0.00%	*p* < 0.001	*p* > 0.999	
AST Guideline	CLSI	67 (4,179, 7,351)	0.590 (0.528, 0.649)	94.82%	*p* = 0.005	*p* < 0.001	*p* = 0.111
	ND	8 (227, 430)	0.705 (0.388, 0.900)	94.99%	*p* = 0.198	*p* < 0.001	
	Multiple Guideline	1 (26, 60)	0.433 (0.315, 0.560)	0.00%	*p* = 0.303	*p* > 0.999	
	EUCAST	7 (795, 2,712)	0.363 (0.163, 0.625)	99.12%	*p* = 0.304	*p* < 0.001	
AST Method	MIX	9 (736, 1857)	0.668 (0.479, 0.815)	97.25%	*p* = 0.080	*p* < 0.001	p = 0.121
	Disk Diffusion	53 (2,265, 4,274)	0.553 (0.481, 0.622)	93.39%	*p* = 0.151	*p* < 0.001	
	MIC	6 (1,022, 1,601)	0.663 (0.564, 0.750)	89.68%	*p* = 0.002	*p* < 0.001	
	Automate	8 (875, 1,098)	0.748 (0.483, 0.904)	95.80%	*p* = 0.065	*p* < 0.001	
Species	MRSA	23 (1,353, 2,733)	0.637 (0.528, 0.733)	95.45%	*p* = 0.014	*p* < 0.001	*p* = 0.074
	mrCoNS	1 (120, 147)	0.816 (0.745, 0.871)	0.00%	*p* < 0.001	*p* > 0.999	
	*S. lugdunensis*	1 (21, 28)	0.750 (0.561, 0.876)	0.00%	*p* = 0.012	*p* > 0.999	
	*S. aureus*	40 (2,907, 6,072)	0.546 (0.442, 0.645)	97.45%	*p* = 0.387	*p* < 0.001	
	MSSA	3 (157, 533)	0.185 (0.067, 0.417)	94.30%	*p* = 0.011	*p* < 0.001	
	*S. epidermidis*	5 (157, 283)	0.509 (0.338, 0.678)	81.37%	*p* = 0.917	*p* < 0.001	
	CoNS	6 (407, 567)	0.767 (0.571, 0.891)	91.58%	*p* = 0.010	*p* < 0.001	
	*Staphylococcus spp*	4 (105, 190)	0.562 (0.296, 0.797)	88.15%	*p* = 0.660	*p* < 0.001	
Coagulase	CPS	66 (4,417, 9,338)	0.558 (0.485, 0.629)	96.90%	*p* = 0.121	*p* < 0.001	*p* = 0.312
	CoNS	13 (705, 1,025)	0.679 (0.563, 0.777)	88.70%	*p* = 0.003	*p* < 0.001	
	ND	4 (105, 190)	0.562 (0.296, 0.797)	88.15%	*p* = 0.660	*p* < 0.001	
Year Group	2015_2019	44 (3,537, 7,509)	0.584 (0.492, 0.671)	97.55%	*p* = 0.073	*p* < 0.001	*p* = 0.901
	2020_2023	39 (1,690, 3,044)	0.569 (0.483, 0.651)	93.20%	*p* = 0.117	*p* < 0.001	

K: Number of reports, n: Number of resistant isolates, N: Number of total isolates, LCI: 95% Lower Limit Confidence Interval, HCI: 95% Higher Limit Confidence Interval, P1: *p*-value of difference from zero resistance rate, P2: *p*-value of heterogeneity between reports, P3: *p*-value of difference between groups.

#### 3.3.1 Subgroup analysis based on countries

**Figure 4:**
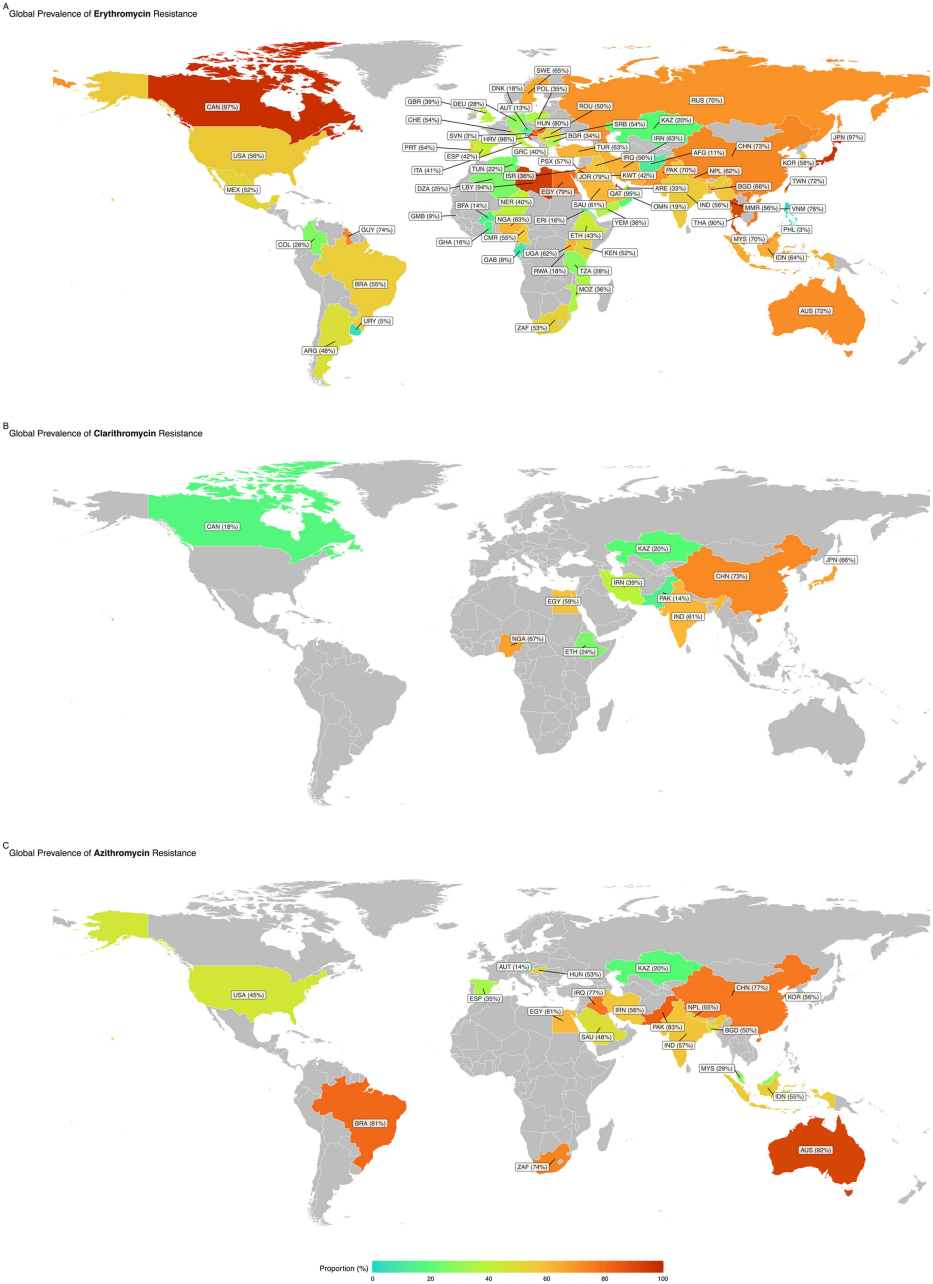
Global prevalence of antibiotic resistance in *Staphylococcus*: maps showing the worldwide prevalence of resistance to Erythromycin **(A)**, Clarithromycin **(B)**, and Azithromycin **(C)**. Regions with higher resistance proportions are highlighted in warmer colors (e.g., red), while areas with lower resistance rates are shown in cooler tones (e.g., green).

Subgroup analysis revealed statistically significant differences in antimicrobial resistance prevalence between countries for azithromycin, clarithromycin, and erythromycin. Austria had the lowest resistance rate for azithromycin, with a prevalence of 13.5%, while Australia had the highest resistance rate at 92.1%. Pakistan had the lowest resistance rate (14.3%) for clarithromycin, while China had the highest (72.9%). The Philippines had the lowest resistance rate of 2.8% for erythromycin, while Canada had the highest resistance rate of 97.4% (Figure 4).

#### 3.3.2 Subgroup analysis based on continents

**Figure 5:**
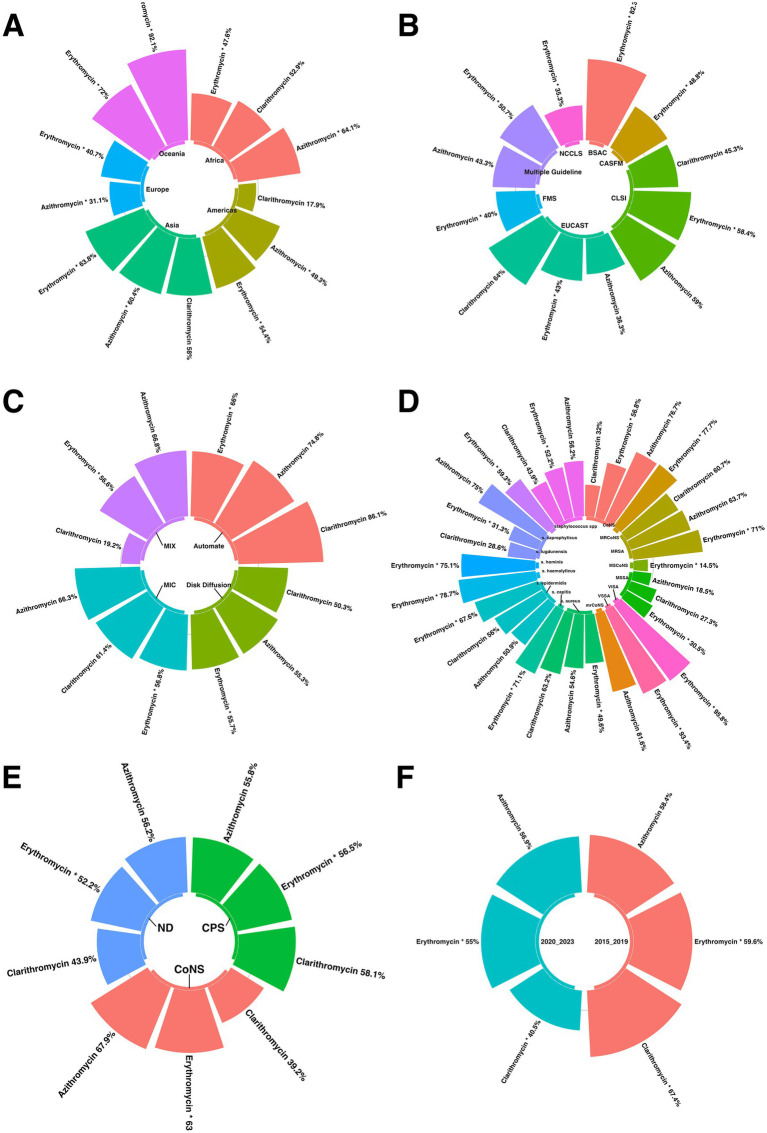
subgroup analysis results were illustrated in figures **(A)** compression of the prevalence of antibiotic-resistant *staphylococcus* isolates between continents; **(B)** Compression of the prevalence of antibiotic-resistant *staphylococcus* isolates between AST guideline; **(C)** Compression of the prevalence of *staphylococcus* isolates AST method; **(D)** Compression of the prevalence of antibiotic-resistant *staphylococcus* isolates based on species **(E)** Compression of the prevalence of antibiotic-resistant *staphylococcus* isolates based on coagulase; **(F)** Compression of the prevalence of *staphylococcus* isolates before and after 2020.

Subgroup analysis revealed statistically significant differences in antimicrobial resistance prevalence between continents, particularly for azithromycin and erythromycin. Europe had the lowest resistance rate for azithromycin, with a prevalence of 31.1%, while Oceania had the highest resistance rate of 92.1%. Similarly, Europe had the lowest resistance rate for erythromycin, with a prevalence of 40.7%, while Oceania had the highest resistance rate at 72% (Figure 5A).

#### 3.3.3 Subgroup analysis based on AST guideline

The subgroup analysis identified statistically significant differences in antibiotic resistance prevalence, including erythromycin, between different antimicrobial susceptibility testing (AST) guidelines. For erythromycin, the NCCLS guideline showed the lowest resistance rate with a prevalence of 35.3%, while the BSAC guideline showed the highest resistance rate at 82.3% (Figure 5B).

#### 3.3.4 Subgroup analysis based on the AST method

Subgroup analysis revealed a statistically significant disparity in the prevalence of antibiotic resistance, including erythromycin, among the various AST methods. For erythromycin, the AST method with the lowest resistance rate was Disk Diffusion, with a prevalence of 55.7%. Conversely, the AST method, with the highest resistance rate, was automated, with a prevalence rate of 66% (Figure 5C).

#### 3.3.5 Subgroup analysis based on species

Subgroup analysis revealed statistically significant differences in antibiotic resistance prevalence among different species, including erythromycin. For erythromycin, MSCoNS had the lowest resistance rate with a prevalence of 14.5%, while VISA had the highest resistance rate with a prevalence of 95.8% (Figure 5D).

#### 3.3.6 Subgroup analysis based on coagulase

Subgroup analysis revealed statistically significant differences in the prevalence of antibiotic resistance, including erythromycin, among different coagulase types. For erythromycin, the coagulase type with the lowest resistance rate was ND, with a prevalence of 52.2%. In contrast, the highest resistance rate was observed for CoNS, with a prevalence of 63.2% (Figure 5E).

#### 3.3.7 Subgroup analysis based on year-group

The subgroup analysis identified statistically significant differences in antibiotic resistance prevalence among different groups, including clarithromycin and erythromycin. For clarithromycin, the period with the lowest resistance rate was 2020–2023, with a prevalence of 40.5%, while the highest resistance rate was observed in 2015–2019, with a prevalence of 67.4%. Similarly, for erythromycin, the lowest resistance rate occurred during 2020–2023, with a prevalence of 55%, while the highest resistance rate was observed during 2015–2019, with a prevalence of 59.6% (Figure 5F).

### 3.4 Meta-regression

**Figure 6:**
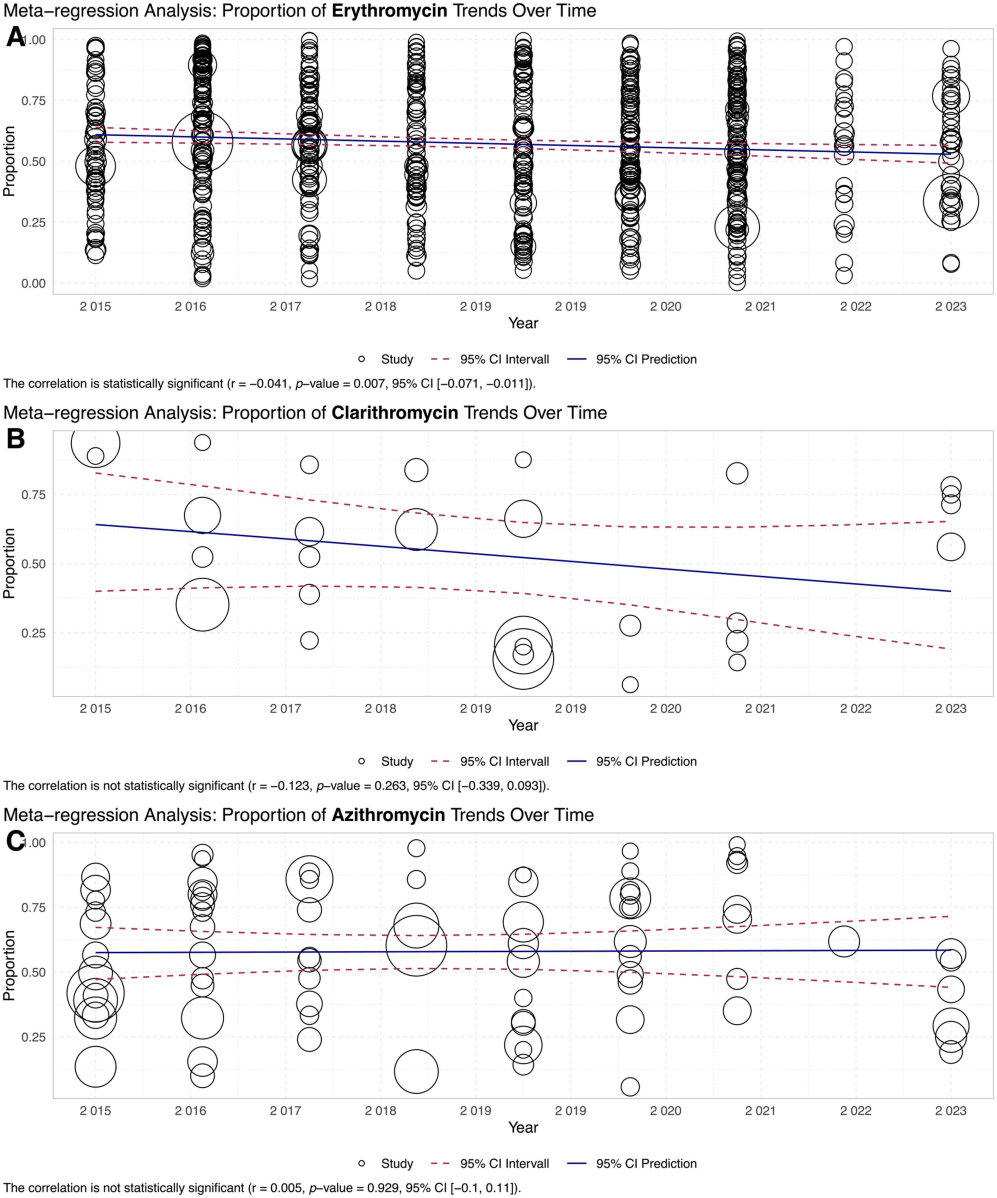
Trends in antibiotic resistance over time (2015–2023): meta-regression analysis plots the trends of resistance proportions for: **(A)** Erythromycin, showing a slight but statistically significant decline. **(B)** Clarithromycin, demonstrating a non-significant downward trend. **(C)** Azithromycin, with no significant trend observed. Data points represent study-specific resistance proportions over time, with bubble sizes reflecting sample size.

Meta-regression analysis was performed to examine the relationship between antimicrobial resistance rates and year of reporting. No statistically significant correlation was observed for erythromycin (r = −0.041, *p*-value = 0.007, 95% CI [−0.071, −0.011]) (Figure 6A). Similarly, the correlation was not statistically significant for clarithromycin (r = −0.123, *p*-value = 0.263, 95% CI [−0.339, 0.093]) (Figure 6B). These results suggest that resistance rates for azithromycin and clarithromycin remained relatively stable over the study period. In contrast, a statistically significant positive correlation was observed for azithromycin (r = 0.005, *p*-value = 0.929, 95% CI [−0.1, 0.11]) (Figure 6C), indicating an upward trend in erythromycin resistance rates over time.

## 4 Discussion

This systematic review and meta-analysis thoroughly evaluated the prevalence and trends of macrolide resistance in *Staphylococcus* species, explicitly focusing on resistance to erythromycin, clarithromycin, and azithromycin. By analyzing data from 207 studies conducted in 76 countries between 2015 and 2023, our findings provide valuable insights into global patterns of macrolide resistance in *Staphylococcus* species. Erythromycin, the first macrolide antibiotic discovered, remains effective in treating minor skin infections caused by penicillin-resistant *S. aureus* strains (Washington and Wilson, 1985). This meta-analysis revealed that erythromycin was the most commonly tested macrolide in antibiotic susceptibility studies, with data from 207 studies in 76 countries. The pooled prevalence of resistance was 57.3%, with significant heterogeneity between studies (I^2^ = 96.09%, *p* < 0.001). Evidence of publication bias was also detected using Egger’s test (*p* < 0.001), resulting in an adjusted pooled prevalence of 50.1% after Fill and Trim analysis. These variations may be due to differences in study populations, periods, sampling methods, or clinical specimen types.

Subgroup analyses revealed significant regional differences in erythromycin resistance rates. Oceania had the highest resistance rate (72%, based on two reports), while Asia contributed the most studies (417 reports) with a pooled prevalence of 63.8%. In particular, China, Iran, and India reported resistance rates of 73.1, 62.7, and 55.7%, respectively, based on 105, 85, and 79 reports. In contrast, Europe had the lowest pooled prevalence of erythromycin-resistant isolates (40.7%, 44 reports), with Spain (13 reports) and Poland (8 reports) reporting prevalence rates of 42.5 and 35%, respectively. The lower resistance rates in Europe reflect increased public awareness and effective public health interventions to curb antimicrobial resistance.

On the other hand, prevalence rates of over 90% for erythromycin-resistant isolates in countries such as Qatar, Canada, Libya, Japan, and Croatia raise significant concerns. However, because these findings are based on AST performed at a single clinical center in each country, the results cannot be generalized to the entire population in these regions. This underscores the need for comprehensive national surveillance systems to monitor antimicrobial resistance in these areas.

Subgroup analysis by species revealed a pooled prevalence of erythromycin resistance in 49.6% of *S. aureus* isolates (342 reports). In addition, some studies included in this meta-analysis reported erythromycin resistance rates for *S. aureus* in two subgroups: MSSA (methicillin-susceptible *S. aureus*) and MRSA. The prevalence of resistance was significantly higher in MRSA than in MSSA (71% vs. 30.5%). However, more studies have focused on MRSA than MSSA (212 vs. 37). These findings are consistent with other meta-analyses that have reported pooled prevalence rates of erythromycin-resistant *S. aureus* isolates (Eshetie et al., 2016; Khanal et al., 2021; Chelkeba et al., 2022; Chelkeba and Melaku, 2022; Ezeh et al., 2023; Xu et al., 2024). However, most of these studies were based on data from one African country and had fewer studies than ours. Moreover, Chelkeba et al. (2022) and Chelkeba and Melaku (2022), during two separate meta-analyses conducted in Ethiopia, reported 50 and 45% prevalence rates for erythromycin-resistant *S. aureus* isolates in women with bacteriuria and patients with wound infections, respectively. In a meta-analysis review, Ezeh et al. (2023) reported a prevalence rate of 47% for erythromycin-resistant *S. aureus* isolates in Nigeria (66 reports) up to 2022. However, data from our meta-analysis highlighted a higher prevalence of erythromycin resistance in Nigeria (23 reports) than in Ezeh et al. (2023) (62.6% vs. 47%). The observed discrepancy in prevalence rates may be due to differences in the periods and number of studies included in these two meta-analyses. Subgroup analysis by species revealed a high pooled prevalence of erythromycin resistance among CoNS isolates at 56.8% (based on 42 reports). In addition, some studies independently reported the frequency of specific CoNS species, allowing pooled prevalence rates to be calculated for each species. Among these, *S. epidermidis* was the most commonly studied CoNS species (41 reports), with a pooled erythromycin resistance prevalence of 67.7%. Similar to our findings, Deyno et al. (2018) also reviewed the prevalence of antimicrobial resistance among clinical isolates of CoNS in Ethiopia through 2016, reporting a 30% prevalence of erythromycin-resistant CoNS. The discrepancy between our findings and Deyno et al. (2018) may be due to differences in the periods and geographic regions covered by these two meta-analyses. Specifically, our meta-analysis included data collected between 2015 and 2023, whereas Deyno et al. (2018) focused on data up to 2016. Furthermore, our study provided a global overview of antimicrobial resistance prevalence, whereas Deyno et al. (2018) limited their analysis to Ethiopia.

In addition, five studies reported a resistance prevalence of 77.7% among [methicillin-resistant Coagulase-Negative *Staphylococci* (MRCoNS)], which was significantly higher than the 14.5% reported in a single survey of MSCoNS. However, due to the unequal number of studies, this comparison lacks balance, and further research is needed to make a comprehensive and accurate comparison.

Overall, the prevalence of MRCoNS was significantly lower than that of MRSA. This difference may be attributed to the lower frequency of CoNS infections than *S. aureus* infections, reducing antimicrobial exposure. However, CoNS have transitioned from being non-pathogenic to emerging as pathogenic strains, potentially acquiring resistance genes from *S. aureus* (Yu et al., 2017).

In contrast, the prevalence of erythromycin resistance decreased slightly over time, from 59.6% in 2015–2019 to 55% in 2020–2023. This decline may reflect increased national efforts to combat antimicrobial resistance and the implementation of updated treatment guidelines and surveillance systems in developed countries. Similarly, a meta-analysis by Xu et al. (2024), found no significant change in erythromycin-resistant *S. aureus* isolates from Cystic fibrosis patients when comparing the periods 2008–2015 and 2015–2021.

Based on AST guidelines, the subgroup analysis showed higher resistance levels in the CLSI group compared to the EUCAST group (58.4% vs. 43%). However, this finding may be influenced by more studies using CLSI guidelines (563) compared to EUCAST guidelines (67 studies). Both guidelines are widely used but differ in their breakpoints for determining resistance. For example, EUCAST defines resistance as MIC >1, whereas CLSI uses MIC ≥8. Similarly, EUCAST considers a zone diameter of <21 mm resistant, while CLSI uses a zone diameter of ≤13 mm. These differences and variations in the number of studies likely contributed to the observed differences in erythromycin resistance prevalence.

This meta-analysis found fewer studies evaluated susceptibility testing for azithromycin and clarithromycin than erythromycin. It may be due to the limited clinical use of azithromycin and clarithromycin for treating staphylococcal infections compared to erythromycin. The pooled prevalence of azithromycin resistance was similar to that of erythromycin (57.3% vs. 57.9%). However, significant heterogeneity between studies was observed (I^2^ = 96.5%, *p* < 0.001), and Egger’s test indicated potential publication bias (*p* < 0.001). After applying fill and trim analysis, the pooled prevalence of azithromycin resistance was adjusted to 51.9%.

The highest resistance rates were reported in Oceania (92.1%, based on one report), while most studies (58 reports) were conducted in Asia, with a pooled prevalence of 60.4%. Specifically, India and Iran contributed 17 and 11 reports, respectively, with 57.5 and 56.3% resistance prevalence rates. Like erythromycin, Europe had the lowest prevalence of azithromycin resistance (31.1%, based on six studies). This low prevalence may be due to the limited number of European studies and the infrequent use of azithromycin to treat staphylococcal infections in this region. Alarmingly, high levels of azithromycin-resistant isolates were identified in Pakistan, Brazil, and China.

Subgroup analysis by species showed that *S. aureus* was the most commonly studied species, with a pooled resistance prevalence of 54.6% (40 reports). In addition, 23 studies reported a high prevalence of azithromycin resistance among MRSA isolates (63.7%), compared with only three studies evaluating MSSA isolates, which showed a much lower resistance prevalence of 18.5%. However, this comparison was biased due to the unequal number of studies. Subgroup analysis by the AST method showed that disc diffusion was the most commonly used method for antibiotic susceptibility testing, probably because of its accessibility and widespread acceptance. However, the highest prevalence of azithromycin resistance was associated with the automated method (74.8%, based on eight reports). Like erythromycin, the prevalence of azithromycin resistance decreased slightly over time, from 58.4% in 2015–2019 to 56.9% in 2020–2023.

Clarithromycin was the third macrolide antibiotic studied in this meta-analysis, with a pooled resistance prevalence of 52.6%; however, there was considerable heterogeneity between studies (I^2^ = 98.76%, *p* < 0.001). Most of the reports (17) were from Asia, with a pooled prevalence of 58%. *S. aureus* was the dominant species, with a resistance prevalence of 63.2%; six studies showed a prevalence rate of 60.7% among MRSA isolates and 27.3% among MSSA isolates (two reports). In contrast to erythromycin and azithromycin, the prevalence of resistance to clarithromycin decreased significantly over different periods (67.4% from 2015 to 2019 and 40.5% from 2020 to 2023).

Clarithromycin, the third macrolide antibiotic examined in this meta-analysis, had a pooled resistance prevalence of 52.6%, although significant heterogeneity between studies was observed (I^2^ = 98.76%, *p* < 0.001). Most reports (17 studies) were from Asia, with a pooled resistance prevalence of 58%. *S. aureus* was the predominant species, with a resistance prevalence of 63.2%. Among MRSA isolates, six studies reported a resistance prevalence of 60.7%, while MSSA isolates had a lower prevalence of 27.3% (based on two reports). In contrast to erythromycin and azithromycin, clarithromycin resistance decreased significantly over time, from 67.4% in 2015–2019 to 40.5% in 2020–2023.

This meta-analysis is the first to compare the prevalence of resistance to azithromycin and clarithromycin in *Staphylococcus* species. As a result, no previous meta-analyses have provided comparable global results.

A significant limitation of this study is the lack of differentiation between *Staphylococcus* species isolated from healthcare and community settings. This distinction is critical, as antibiotic resistance rates in healthcare settings are typically higher than in the community. Another limitation is the lack of data on resistance to newer macrolides, primarily due to the limited number of studies investigating them. This gap highlights the need for further research to provide accurate and comprehensive evidence.

## 5 Conclusion

This meta-analysis highlights a relatively high prevalence of macrolide resistance in *S. aureus* and CoNS isolates worldwide. These elevated resistance rates underscore the importance of regular epidemiologic surveillance of antimicrobial resistance and the implementation of stewardship programs. Most of the studies included in this analysis were conducted in Asia, while Europe had the lowest macrolide resistance rate. In addition, resistance to erythromycin and azithromycin remained relatively stable between 2015–2019 and 2020–2023. Nevertheless, antimicrobial susceptibility testing before treatment is recommended, and further research into the molecular and genetic mechanisms of macrolide resistance is strongly encouraged.

## Data Availability

The original contributions presented in the study are included in the article/supplementary material, further inquiries can be directed to the corresponding authors.

## References

[ref1] Abbasi M. BaseriSalehi M. Bahador N. Taherikalani M. (2017). Antibiotic resistance patterns and virulence determinants of different SCCmec and Pulsotypes of *Staphylococcus Aureus* isolated from a major Hospital in Ilam, Iran. Open Microbiol. J. 11, 211–223. doi: 10.2174/1874285801711010211, PMID: 29204221 PMC5688384

[ref2] Abdulmanea A. A. Alharbi N. S. Somily A. M. Khaled J. M. Algahtani F. H. (2023). The prevalence of the virulence genes of *Staphylococcus aureus* in sickle cell disease patients at KSUMC, Riyadh, Saudi Arabia. Antibiotics (Basel) 12:1221. doi: 10.3390/antibiotics12071221, PMID: 37508317 PMC10416153

[ref3] Abebe A. A. Birhanu A. G. (2023). Methicillin resistant *Staphylococcus aureus*: molecular mechanisms underlying drug resistance development and novel strategies to combat. Infect Drug. Resist. 16, 7641–7662. doi: 10.2147/IDR.S428103, PMID: 38111667 PMC10726795

[ref4] Abouelnour A. Zaki M. Hassan R. Elkannishy S. (2019). Phenotypic and genotypic identification of *Staphylococcus aureus* resistant to clindamycin in Mansoura university children hospital, Egypt. Afr. J. Clin. Exp. Microbiol. 21, 30–35. doi: 10.4314/ajcem.v21i1.4

[ref5] Ackers-Johnson G. Kibombo D. Kusiima B. Nsubuga M. L. Kigozi E. Kajumbula H. M. . (2021). Antibiotic resistance profiles and population structure of disease-associated *Staphylococcus aureus* infecting patients in Fort Portal regional referral hospital, Western Uganda. Microbiology (Reading) 167:001000. doi: 10.1099/mic.0.001000, PMID: 34032566 PMC8290103

[ref6] Adhikari P. Basyal D. Rai J. R. Bharati L. Budthapa A. Gharti K. P. . (2023). Prevalence, antimicrobial susceptibility pattern and multidrug resistance of methicillin-resistant *Staphylococcus aureus* isolated from clinical samples at a tertiary care teaching hospital: an observational, cross-sectional study from the Himalayan country, Nepal. BMJ Open 13:e067384. doi: 10.1136/bmjopen-2022-067384, PMID: 37164471 PMC10174000

[ref7] Agarwal L. Singh A. K. Agarwal A. Agarwal A. (2016). Methicillin and mupirocin resistance in nasal colonizers coagulase-negative Staphylococcus among health care workers. Med. J. Dr. DY Patil Univ. 9, 479–483. doi: 10.4103/0975-2870.186070

[ref8] Aguinagalde L. Diez-Martinez R. Yuste J. Royo I. Gil C. Lasa I. . (2015). Auranofin efficacy against MDR Streptococcus pneumoniae and *Staphylococcus aureus* infections. J. Antimicrob. Chemother. 70, 2608–2617. doi: 10.1093/jac/dkv163, PMID: 26142477

[ref9] Ahangarzadeh Rezaee M. Mirkarimi S. F. Hasani A. Sheikhalizadeh V. Soroush M. H. Abdinia B. (2016). Molecular typing of *Staphylococcus aureus* isolated from clinical specimens during an eight-year period (2005–2012) in Tabriz, Iran. Arch. Pediatr. Infect. Dis. 4:e35563. doi: 10.5812/pedinfect.35563

[ref10] Ahmad M. Kumar P. Sultan A. Akhtar A. Chaudhary B. Khan F. (2020). Prevalence of community acquired Uropathogens and their antimicrobial susceptibility in patients from the urology unit of a tertiary care medical center. J. Pure Appl. Microbiol. 14, 2009–2015. doi: 10.22207/JPAM.14.3.40

[ref11] Akbariyeh H. Nahaei M. R. Hasani A. Pormohammad A. (2017). Intrinsic and acquired methicillin-resistance detection in Staphylococcus aureus and its relevance in therapeutics. Arch. Pediatr. Infect. Dis. 5:e39185. doi: 10.5812/pedinfect.39185

[ref12] Akpaka P. E. Roberts R. Monecke S. (2017). Molecular characterization of antimicrobial resistance genes against *Staphylococcus aureus* isolates from Trinidad and Tobago. J. Infect. Public Health 10, 316–323. doi: 10.1016/j.jiph.2016.05.010, PMID: 27328777

[ref13] Al Zebary M. K. Yousif S. Y. Assafi M. S. (2017). The prevalence, molecular characterization and antimicrobial susceptibility of isolated from impetigo cases in Duhok, Iraq. Open Dermatol. J. 11, 22–29. doi: 10.2174/1874372201711010022

[ref14] Al-Habsi T. H. A. Al-Lamki R. N. A. Mabruk M. (2020). Antibiotic susceptibility pattern of bacterial isolates from wound infections among patients attending a tertiary care hospital in Oman. Biomed. Pharma. J. 13, 2069–2080. doi: 10.13005/bpj/2087

[ref15] Al-Humaidan O. S. El-Kersh T. A. Al-Akeel R. A. (2015). Risk factors of nasal carriage of Staphylococcus aureus and methicillin-resistant *Staphylococcus aureus* among health care staff in a teaching hospital in Central Saudi Arabia. Saudi Med. J. 36, 1084–1090. doi: 10.15537/smj.2015.9.12460, PMID: 26318466 PMC4613633

[ref16] Almasri M. Abu Hasan N. Sabbah N. (2016). Macrolide and lincosamide resistance in staphylococcal clinical isolates in Nablus, Palestine. Turk. J. Med. Sci. 46, 1064–1070. doi: 10.3906/sag-1503-121, PMID: 27513405

[ref17] Almohammady M. N. Eltahlawy E. M. Reda N. M. (2020). Pattern of bacterial profile and antibiotic susceptibility among neonatal sepsis cases at Cairo University children hospital. J. Taibah Univ. Med. Sci. 15, 39–47. doi: 10.1016/j.jtumed.2019.12.005, PMID: 32110181 PMC7033391

[ref18] Al-Naqshbandi A. A. Chawsheen M. A. Abdulqader H. H. (2019). Prevalence and antimicrobial susceptibility of bacterial pathogens isolated from urine specimens received in rizgary hospital—Erbil. J. Infect. Public Health 12, 330–336. doi: 10.1016/j.jiph.2018.11.005, PMID: 30522892

[ref19] Al-Qaisi M. M. Al-Salmani T. S. (2020). Phenotypic detection of macrolide-Lincosamide-Streptogramin resistance among Staphylococcus aureus and *Staphylococcus epidermidis* in Baghdad, Iraq. Int. J. Drug Deliv. Technol. 10, 431–436. doi: 10.25258/ijddt.10.3.22

[ref20] AL-Salihi S. S. Karim G. F. Al-Bayati A. Obaid H. M. (2023). Prevalence of methicillin-resistant and methicillin sensitive *Staphylococcus aureus* nasal carriage and their antibiotic resistant patterns in Kirkuk City, Iraq. J. Pure Appl. Microbiol. 17, 329–337. doi: 10.22207/JPAM.17.1.22

[ref21] Al-Tamimi M. Himsawi N. Abu-Raideh J. Khasawneh A. I. Jazar D. A. Al-Jawaldeh H. . (2021). Phenotypic and molecular screening of Nasal *S. aureus* from adult hospitalized patients for methicillin- and vancomycin-resistance. Infect. Disord. Drug Targets 21, 68–77. doi: 10.2174/1871526520666200109143158, PMID: 31916522

[ref22] Al-Taweel R. S. (2020). Bacterial contamination of stethoscopes. Biochem. Cell. Arch. 20:6187.

[ref23] An N. V. Hai L. H. L. Luong V. H. Vinh N. T. H. Hoa P. Q. Hung L. V. . (2024). Antimicrobial resistance patterns of *Staphylococcus Aureus* isolated at a general Hospital in Vietnam between 2014 and 2021. Infect. Drug Resist. 17, 259–273. doi: 10.2147/IDR.S437920, PMID: 38283112 PMC10822110

[ref24] Arabestani M. R. Rastiyani S. Alikhani M. Y. Mousavi S. F. (2018). The relationship between prevalence of antibiotics resistance and virulence factors genes of MRSA and MSSA strains isolated from clinical samples, West Iran. Oman Med. J. 33, 134–140. doi: 10.5001/omj.2018.25, PMID: 29657682 PMC5889837

[ref25] Asaad A. M. Ansar Qureshi M. Mujeeb Hasan S. (2016). Clinical significance of coagulase-negative staphylococci isolates from nosocomial bloodstream infections. Infect Dis. (Lond) 48, 356–360. doi: 10.3109/23744235.2015.1122833, PMID: 26666168

[ref26] Asbell P. A. Sanfilippo C. M. Pillar C. M. DeCory H. H. Sahm D. F. Morris T. W. (2015). Antibiotic resistance among ocular pathogens in the United States five-year results from the antibiotic resistance monitoring in ocular microorganisms (ARMOR) surveillance study. JAMA Ophthalmol. 133, 1445–1454. doi: 10.1001/jamaophthalmol.2015.3888, PMID: 26502312

[ref27] Baek Y. S. Jeon J. Ahn J. W. Song H. J. (2016). Antimicrobial resistance of *Staphylococcus aureus* isolated from skin infections and its implications in various clinical conditions in Korea. Int. J. Dermatol. 55, e191–e197. doi: 10.1111/ijd.13046, PMID: 26892888

[ref28] Bai B. Lin Z. Pu Z. Xu G. Zhang F. Chen Z. . (2019). In vitro activity and Heteroresistance of Omadacycline against clinical *Staphylococcus aureus* isolates from China reveal the impact of Omadacycline susceptibility by branched-chain amino acid transport system II carrier protein, Na/pi cotransporter family protein, and fibronectin-binding protein. Front. Microbiol. 10:2546. doi: 10.3389/fmicb.2019.02546 31787948 PMC6856048

[ref29] Banawas S. S. Alobaidi A. S. Dawoud T. M. AlDehaimi A. Alsubaie F. M. Abdel-Hadi A. . (2023). Prevalence of multidrug-resistant Bacteria in healthcare-associated bloodstream infections at hospitals in Riyadh, Saudi Arabia. Pathogens 12:1075. doi: 10.3390/pathogens12091075, PMID: 37764883 PMC10536600

[ref30] Barcudi D. Sosa E. J. Lamberghini R. Garnero A. Tosoroni D. Decca L. . (2020). MRSA dynamic circulation between the community and the hospital setting: new insights from a cohort study. J. Infect. 80, 24–37. doi: 10.1016/j.jinf.2019.10.001, PMID: 31606351

[ref31] Baz A. A. Bakhiet E. K. Abdul-Raouf U. Abdelkhalek A. (2021). Prevalence of enterotoxin genes (SEA to SEE) and antibacterial resistant pattern of *Staphylococcus aureus* isolated from clinical specimens in Assiut city of Egypt. Egyptian J. Med. Hum. Genet. 22, 1–12. doi: 10.1186/s43042-021-00199-0

[ref32] Begg C. B. Mazumdar M. (1994). Operating characteristics of a rank correlation test for publication bias. Biometrics 50, 1088–1101. doi: 10.2307/2533446, PMID: 7786990

[ref33] Belbase A. Pant N. D. Nepal K. Neupane B. Baidhya R. Baidya R. . (2017). Antibiotic resistance and biofilm production among the strains of *Staphylococcus aureus* isolated from pus/wound swab samples in a tertiary care hospital in Nepal. Ann. Clin. Microbiol. Antimicrob. 16:15. doi: 10.1186/s12941-017-0194-0, PMID: 28330484 PMC5363015

[ref34] Belete M. A. (2020). Bacterial profile and ESBL screening of urinary tract infection among asymptomatic and symptomatic pregnant women attending antenatal Care of Northeastern Ethiopia Region. Infect Drug Resist. 13, 2579–2592. doi: 10.2147/IDR.S258379, PMID: 32801795 PMC7395684

[ref35] Bensaci M. Sahm D. (2017). Surveillance of tedizolid activity and resistance: in vitro susceptibility of gram-positive pathogens collected over 5 years from the United States and Europe. Diagn. Microbiol. Infect. Dis. 87, 133–138. doi: 10.1016/j.diagmicrobio.2016.10.009, PMID: 27866673

[ref36] Bhatt P. Tandel K. Singh A. Mugunthan M. Grover N. Sahni A. K. (2016). Species distribution and antimicrobial resistance pattern of coagulase-negative staphylococci at a tertiary care Centre. Med. J. Armed Forces India 72, 71–74. doi: 10.1016/j.mjafi.2014.12.007, PMID: 26900227 PMC4723702

[ref37] Bhattacharya S. Pal K. Jain S. Chatterjee S. S. Konar J. (2016). Surgical site infection by methicillin resistant *Staphylococcus aureus*- on decline? J. Clin. Diagn. Res. 10, DC32–DC36. doi: 10.7860/JCDR/2016/21664.8587, PMID: 27790436 PMC5071936

[ref38] Bhavana A. M. Kumari P. H. P. Mohan N. Chandrasekhar V. Vijayalakshmi P. Manasa R. V. (2019). Bacterial vaginosis and antibacterial susceptibility pattern of asymptomatic urinary tract infection in pregnant women at a tertiary care hospital, Visakhaptn, India. Iran J. Microbiol. 11, 488–495, PMID: 32148680 PMC7048967

[ref39] Biset S. Moges F. Endalamaw D. Eshetie S. (2020). Multi-drug resistant and extended-spectrum beta-lactamases producing bacterial uropathogens among pregnant women in Northwest Ethiopia. Ann. Clin. Microbiol. Antimicrob. 19:25. doi: 10.1186/s12941-020-00365-z, PMID: 32493343 PMC7268622

[ref40] Bishr A. S. Abdelaziz S. M. Yahia I. S. Yassien M. A. Hassouna N. A. Aboshanab K. M. (2021). Association of Macrolide Resistance Genotypes and Synergistic Antibiotic Combinations for combating macrolide-resistant MRSA recovered from hospitalized patients. Biology (Basel) 10. doi: 10.3390/biology10070624, PMID: 34356479 PMC8301042

[ref41] Bolatchiev A. (2020). Antibacterial activity of human defensins against Staphylococcus aureus and *Escherichia coli*. PeerJ 8:e10455. doi: 10.7717/peerj.10455, PMID: 33304659 PMC7698690

[ref42] Boncompain C. A. Suarez C. A. Squeff M. Belluzo V. Piccirilli G. Molteni A. . (2023). Phenotypic and molecular characterization of *Staphylococcus aureus* isolates conducted in nares of psoriatic patients attending a public hospital in Argentina. Rev. Argent. Microbiol. 55, 3–11. doi: 10.1016/j.ram.2022.02.008, PMID: 35760653

[ref43] Cavalcante F. S. Alvarenga C. Saintive S. Dios E. Carvalho D. Netto K. (2020). *Staphylococcus aureus* nasal isolates may have the same genetic profile in atopic dermatitis paediatric patients and their close contacts. J. Med. Microbiol. 69, 850–853. doi: 10.1099/jmm.0.001197, PMID: 32430095

[ref44] Cavanagh J. P. Wolden R. Heise P. Esaiassen E. Klingenberg C. Aarag Fredheim E. G. (2016). Antimicrobial susceptibility and body site distribution of community isolates of coagulase-negative staphylococci. APMIS 124, 973–978. doi: 10.1111/apm.12591, PMID: 27599662

[ref45] Chaleshtori S. H. Kachoie M. A. (2016). Chemical composition and antimicrobial effects of *calendula officinalis* grown under chemical and biological conditions on the methicillin-resistant *staphylococcus aureus* isolated from hospital infections. Biosci. Biotechnol. Res. Asia 13, 1787–1796. doi: 10.13005/bbra/2331

[ref46] Changchien C. H. Chen S. W. Chen Y. Y. Chu C. (2016). Antibiotic susceptibility and genomic variations in *Staphylococcus aureus* associated with skin and soft tissue infection (SSTI) disease groups. BMC Infect. Dis. 16:276. doi: 10.1186/s12879-016-1630-z, PMID: 27287530 PMC4902997

[ref47] Chauhan S. Surender Rappai T. (2021). Mupirocin resistance in *staphylococcus aureus* isolated from nasal swabs of ICU and OT staff- a study from a tertiary care hospital. J Pure Appl Microbiol 15, 2059–2064. doi: 10.22207/JPAM.15.4.28

[ref48] Chelkeba L. Fanta K. Mulugeta T. Melaku T. (2022). Bacterial profile and antimicrobial resistance patterns of common bacteria among pregnant women with bacteriuria in Ethiopia: a systematic review and meta-analysis. Arch. Gynecol. Obstet. 306, 663–686. doi: 10.1007/s00404-021-06365-4, PMID: 35032208 PMC9411254

[ref49] Chelkeba L. Melaku T. (2022). Epidemiology of staphylococci species and their antimicrobial-resistance among patients with wound infection in Ethiopia: a systematic review and meta-analysis. J. Glob. Antimicrobial Resist. 29, 483–498. doi: 10.1016/j.jgar.2021.10.025, PMID: 34801740

[ref50] Chen P. Y. Chuang Y. C. Wang J. T. Sheng W. H. Chen Y. C. Chang S. C. (2021). Sequence type 8 as an emerging clone of methicillin-resistant *Staphylococcus aureus* causing bloodstream infections in Taiwan. Emerg Microbes Infect 10, 1908–1918. doi: 10.1080/22221751.2021.1981158, PMID: 34520335 PMC8475108

[ref51] Chen Y. L. Kang E. Y. Yeh L. K. Ma D. H. K. Tan H. Y. Chen H. C. . (2021). Clinical features and molecular characteristics of methicillin-susceptible *Staphylococcus aureus* ocular infection in Taiwan. Antibiotics (Basel) 10. doi: 10.3390/antibiotics10121445, PMID: 34943657 PMC8698105

[ref52] Chen P. Sun F. Feng W. Hong H. Li B. Song J. (2021). Pathogenic characteristics Of*staphylococcus aureus*isolates from arthroplasty infections. Int. J. Artif. Organs 44, 208–214. doi: 10.1177/0391398820948877, PMID: 32783484

[ref53] Cheung G. Y. C. Bae J. S. Otto M. (2021). Pathogenicity and virulence of *Staphylococcus aureus*. Virulence 12, 547–569. doi: 10.1080/21505594.2021.1878688, PMID: 33522395 PMC7872022

[ref54] Choi E. Y. Han J. Y. Lee H. Lee S. C. Koh H. J. Kim S. S. . (2019). Impact of antibiotic resistance of pathogens and early vitrectomy on the prognosis of infectious endophthalmitis: a 10-year retrospective study. Graefes Arch. Clin. Exp. Ophthalmol. 257, 805–813. doi: 10.1007/s00417-019-04261-x, PMID: 30761407

[ref55] Cochran W. G. (1954). The combination of estimates from different experiments. Biometrics 10, 101–129. doi: 10.2307/3001666

[ref56] Conceicao T. de Lencastre H. Aires-de-Sousa M. (2021). Prevalence of biocide resistance genes and chlorhexidine and mupirocin non-susceptibility in Portuguese hospitals during a 31-year period (1985-2016). J. Glob. Antimicrob. Resist. 24, 169–174. doi: 10.1016/j.jgar.2020.12.010, PMID: 33373736

[ref57] Coombs G. W. Daley D. A. Mowlaboccus S. Lee Y. T. Pang S. R. Australian Group on Antimicrobial (2020). Australian group on antimicrobial resistance (AGAR) Australian *Staphylococcus aureus* Sepsis outcome Programme (ASSOP) annual report 2018. Commun. Dis. Intell. 44:44. doi: 10.33321/cdi.2020.44.18, PMID: 32178604

[ref58] Dayie N. Osei M. M. Opintan J. A. Tetteh-Quarcoo P. B. Kotey F. C. N. Ahenkorah J. . (2021). Nasopharyngeal carriage and antimicrobial susceptibility profile of *Staphylococcus aureus* among children under five years in Accra. Pathogens 10. doi: 10.3390/pathogens10020136, PMID: 33572983 PMC7912391

[ref59] de Benito S. Alou L. Becerro-de-Bengoa-Vallejo R. Losa-Iglesias M. E. Gomez-Lus M. L. Collado L. . (2018). "prevalence of Staphylococcus spp. nasal colonization among doctors of podiatric medicine and associated risk factors in Spain." Antimicrob resist. Infect. Control. 7:24. doi: 10.1186/s13756-018-0318-0 PMC5816397 29468052

[ref60] Demir C. Demirci M. Yigin A. Tokman H. B. Cetik Yildiz S. (2020). Presence of biofilm and adhesin genes in *Staphylococcus aureus* strains taken from chronic wound infections and their genotypic and phenotypic antimicrobial sensitivity patterns. Photodiagn. Photodyn. Ther. 29:101584. doi: 10.1016/j.pdpdt.2019.101584, PMID: 31689511

[ref61] Deyno S. Fekadu S. Seyfe S. (2018). Prevalence and antimicrobial resistance of coagulase negative staphylococci clinical isolates from Ethiopia: a meta-analysis. BMC Microbiol. 18:43. doi: 10.1186/s12866-018-1188-6, PMID: 29801462 PMC5970528

[ref62] Dilnessa T. Bitew A. (2016). Prevalence and antimicrobial susceptibility pattern of methicillin resistant *Staphylococcus aureus* isolated from clinical samples at Yekatit 12 hospital medical college, Addis Ababa, Ethiopia. BMC Infect. Dis. 16:398. doi: 10.1186/s12879-016-1742-5, PMID: 27506613 PMC4977752

[ref63] Diriba K. Kassa T. Alemu Y. Bekele S. (2020). In vitro biofilm formation and antibiotic susceptibility patterns of Bacteria from suspected external eye infected patients attending ophthalmology clinic, Southwest Ethiopia. Int J Microbiol 2020:8472395. doi: 10.1155/2020/8472395 32318110 PMC7155758

[ref64] Dormanesh B. Siroosbakhat S. Khodaverdi Darian E. Afsharkhas L. (2015). Methicillin-resistant *Staphylococcus aureus* isolated from various types of hospital infections in pediatrics: Panton-valentine Leukocidin, staphylococcal chromosomal cassette mec SCCmec phenotypes and antibiotic resistance properties. Jundishapur J. Microbiol. 8:e11341. doi: 10.5812/jjm.11341, PMID: 26862375 PMC4741056

[ref65] Doss R. W. Mostafa A. M. A. Arafa A. E. E.-D. Radi N. A. E.-M. (2017). Relationship between lipase enzyme and antimicrobial susceptibility of *Staphylococcus aureus*-positive and *Staphylococcus epidermidis*-positive isolates from acne vulgaris. J. Egypt. Women Dermatol. Soc. 14, 167–172. doi: 10.1097/01.EWX.0000516051.01553.99

[ref66] Duncan L. R. Sader H. S. Flamm R. K. Jones R. N. Mendes R. E. (2016). Oritavancin in vitro activity against contemporary *Staphylococcus aureus* isolates responsible for invasive community- and healthcare-associated infections among patients in the United States (2013-2014). Diagn. Microbiol. Infect. Dis. 86, 303–306. doi: 10.1016/j.diagmicrobio.2016.07.025, PMID: 27543378

[ref67] Eibach D. Nagel M. Hogan B. Azuure C. Krumkamp R. Dekker D. . (2017). Nasal carriage of *Staphylococcus aureus* among children in the Ashanti region of Ghana. PLoS One 12:e0170320. doi: 10.1371/journal.pone.0170320, PMID: 28107412 PMC5249101

[ref68] El Mammery A. Ramirez de Arellano E. Canada-Garcia J. E. Cercenado E. Villar-Gomara L. Casquero-Garcia V. . (2023). An increase in erythromycin resistance in methicillin-susceptible *Staphylococcus aureus* from blood correlates with the use of macrolide/lincosamide/streptogramin antibiotics. EARS-net Spain (2004-2020). Front. Microbiol. 14:1220286. doi: 10.3389/fmicb.2023.1220286, PMID: 37822743 PMC10562549

[ref69] El-Amir M. I. El-Feky M. A. Abo Elwafa D. A. Abd-Elmawgood E. A. (2019). Rapid diagnosis of neonatal sepsis by PCR for detection of 16S rRNA gene, while blood culture and PCR results were similar in E.Coli-predominant EOS cases. Infect Drug Resist 12, 2703–2710. doi: 10.2147/IDR.S213958, PMID: 31564919 PMC6724612

[ref70] El-Baghdady K. Z. El-Borhamy M. I. Abd El-Ghafar H. A. (2020). Prevalence of resistance and toxin genes in community-acquired and hospital-acquired methicillin-resistant *Staphylococcus aureus* clinical isolates. Iran. J. Basic Med. Sci. 23, 1251–1260. doi: 10.22038/ijbms.2020.40260.9534, PMID: 33149856 PMC7585533

[ref71] El-Kersh T. A. Marie M. A. Al-Sheikh Y. A. Al-Agamy M. H. Al Bloushy A. A. (2016). Prevalence and risk factors of early fecal carriage of enterococcus faecalis and Staphylococcus spp and their antimicrobial resistant patterns among healthy neonates born in a hospital setting in Central Saudi Arabia. Saudi Med. J. 37, 280–287. doi: 10.15537/smj.2016.3.13871, PMID: 26905350 PMC4800892

[ref72] Elzorkany K. M. A. Elbrolosy A. M. Salem E. H. (2019). Methicillin-resistant *Staphylococcus aureus* carriage in hemodialysis vicinity: prevalence and decolonization approach. Indian J Nephrol 29, 282–287. doi: 10.4103/ijn.IJN_56_18, PMID: 31423064 PMC6668309

[ref73] Eshetie S. Tarekegn F. Moges F. Amsalu A. Birhan W. Huruy K. (2016). Methicillin resistant *Staphylococcus aureus* in Ethiopia: a meta-analysis. BMC Infect. Dis. 16:689. doi: 10.1186/s12879-016-2014-0, PMID: 27871245 PMC5117566

[ref74] Esmaeili Benvidi M. Houri H. Ghalavand Z. Nikmanesh B. Azimi H. Samadi R. . (2017). Toxin production and drug resistance profiles of pediatric methicillin-resistant *Staphylococcus aureus* isolates in Tehran. J. Infect. Dev. Ctries. 11, 759–765. doi: 10.3855/jidc.9360, PMID: 31600148

[ref75] Estany-Gestal A. Salgado-Barreira A. Vazquez-Lago J. M. (2024). Antibiotic use and antimicrobial resistance: a global public health crisis. Antibiotics (Basel) 13. doi: 10.3390/antibiotics13090900, PMID: 39335073 PMC11428428

[ref76] Ezeh C. K. Eze C. N. Dibua M. E. U. Emencheta S. C. (2023). A meta-analysis on the prevalence of resistance of *Staphylococcus aureus* to different antibiotics in Nigeria. Antimicrob. Resist. Infect. Control 12:40. doi: 10.1186/s13756-023-01243-x, PMID: 37098614 PMC10127087

[ref77] Farah S. M. Alshehri M. A. Alfawaz T. S. Alasmeri F. A. Alageel A. A. Alshahrani D. A. (2019). Trends in antimicrobial susceptibility patterns in king Fahad Medical City, Riyadh, Saudi Arabia. Saudi Med. J. 40, 252–259. doi: 10.15537/smj.2019.3.23947, PMID: 30834420 PMC6468207

[ref78] Fasihi Y. Saffari F. Kandehkar Ghahraman M. R. Kalantar-Neyestanaki D. (2016). Molecular detection of macrolide and Lincosamide-resistance genes in clinical methicillin-resistant *Staphylococcus aureus* isolates from Kerman, Iran. Archiv. Pediatr. Infect. Dis. 5:e37761. doi: 10.5812/pedinfect.37761

[ref79] Fateh Amirkhiz M. Ahangarzadeh Rezaee M. Hasani A. Aghazadeh M. Naghili B. (2015). SCCmec typing of methicillin-resistant *Staphylococcus aureus*: An eight year experience. Archiv. Pediatr. Infect. Dis. 3:e30632. doi: 10.5812/pedinfect.30632

[ref80] Fateh Dizji P. Khosravy M. Saeedi A. A. Asli M. Sepahvand S. Darvishi M. (2023). Prevalence of clindamycin-resistant *Staphylococcus aureus* induced by macrolide resistance, Iran, 2019-2021. Iran. J. Med. Microbiol. 17, 256–261. doi: 10.30699/ijmm.17.2.256

[ref81] Firoozeh F. Omidi M. Saffari M. Sedaghat H. Zibaei M. (2020). Molecular analysis of methicillin-resistant *Staphylococcus aureus* isolates from four teaching hospitals in Iran: the emergence of novel MRSA clones. Antimicrob. Resist. Infect. Control 9:112. doi: 10.1186/s13756-020-00777-8, PMID: 32680563 PMC7367364

[ref82] Fu Y. Xiong M. Li X. Zhou J. Xiao X. Fang F. . (2020). Molecular characteristics, antimicrobial resistance and virulence gene profiles of *Staphylococcus aureus* isolates from Wuhan, Central China. Infect Drug Resist 13, 2063–2072. doi: 10.2147/IDR.S249988, PMID: 32669859 PMC7335743

[ref83] Gajdacs M. Abrok M. Lazar A. Burian K. (2021). Urinary tract infections in elderly patients: a 10-year study on their epidemiology and antibiotic resistance based on the WHO access, watch, reserve (AWaRe) classification. Antibiotics (Basel) 10:1098. doi: 10.3390/antibiotics10091098 34572680 PMC8467796

[ref84] Garza-Gonzalez E. Morfin-Otero R. Mendoza-Olazaran S. Bocanegra-Ibarias P. Flores-Trevino S. Rodriguez-Noriega E. . (2019). A snapshot of antimicrobial resistance in Mexico. Results from 47 centers from 20 states during a six-month period. PLoS One 14:e0209865. doi: 10.1371/journal.pone.0209865, PMID: 30913243 PMC6435111

[ref85] Getaneh A. Ayalew G. Belete D. Jemal M. Biset S. (2021). Bacterial etiologies of ear infection and their antimicrobial susceptibility pattern at the University of Gondar Comprehensive Specialized Hospital, Gondar, Northwest Ethiopia: a six-year retrospective study. Infect Drug Resist 14, 4313–4322. doi: 10.2147/IDR.S332348, PMID: 34707376 PMC8542893

[ref86] Gitau W. Masika M. Musyoki M. Museve B. Mutwiri T. (2018). Antimicrobial susceptibility pattern of *Staphylococcus aureus* isolates from clinical specimens at Kenyatta National Hospital. BMC. Res. Notes 11:226. doi: 10.1186/s13104-018-3337-2, PMID: 29615129 PMC5883409

[ref87] Goes I. Romero L. C. Turra A. J. Gotardi M. A. Rodrigues T. Santos L. O. . (2021). Prevalence of nasal carriers of methicillin-resistant *Staphylococcus aureus* in primary health care units in Brazil. Rev. Inst. Med. Trop. Sao Paulo 63:e14. doi: 10.1590/s1678-9946202163014, PMID: 33656137 PMC7924983

[ref88] Goudarzi M. Abiri P. Nasirian S. Afshari S. G. (2018). SCCmec and spa typing of *Staphylococcus aureus* strains isolated from patients with urinary tract infection: emergence of spa types t426 and t021 in Iran. Jundishapur J. Microbiol. 11:e62169. doi: 10.5812/jjm.62169

[ref89] Goudarzi M. Tayebi Z. Fazeli M. Miri M. Nasiri M. J. (2020). Molecular characterization, drug resistance and virulence analysis of constitutive and inducible clindamycin resistance *Staphylococcus aureus* strains recovered from clinical samples, Tehran—Iran. Infect Drug Resist 13, 1155–1162. doi: 10.2147/IDR.S251450, PMID: 32368106 PMC7183778

[ref90] Gungor S. Karagoz A. Kocak N. Arslantas T. (2021). Methicillin-resistant *Staphylococcus aureus* in a Turkish hospital: characterization of clonal types and antibiotic susceptibility. J. Infect. Dev. Ctries. 15, 1854–1860. doi: 10.3855/jidc.14963, PMID: 35044943

[ref91] Guo Y. Ding Y. Liu L. Shen X. Hao Z. Duan J. . (2019). Antimicrobial susceptibility, virulence determinants profiles and molecular characteristics of *Staphylococcus epidermidis* isolates in Wenzhou, eastern China. BMC Microbiol. 19:157. doi: 10.1186/s12866-019-1523-6, PMID: 31288755 PMC6617921

[ref92] Guo Y. Song G. Sun M. Wang J. Wang Y. (2020). Prevalence and therapies of antibiotic-resistance in *Staphylococcus aureus*. Front. Cell. Infect. Microbiol. 10:107. doi: 10.3389/fcimb.2020.00107, PMID: 32257966 PMC7089872

[ref93] Guo Y. Wang B. Rao L. Wang X. Zhao H. Li M. . (2021). Molecular characteristics of rifampin-sensitive and -resistant isolates and characteristics of rpoB gene mutations in methicillin-resistant *Staphylococcus aureus*. Infect Drug Resist 14, 4591–4600. doi: 10.2147/IDR.S336200, PMID: 34764656 PMC8576291

[ref94] Hailegiyorgis T. T. Sarhie W. D. Workie H. M. (2018). Isolation and antimicrobial drug susceptibility pattern of bacterial pathogens from pediatric patients with otitis media in selected health institutions, Addis Ababa, Ethiopia: a prospective cross-sectional study. BMC Ear Nose Throat Disord 18:8. doi: 10.1186/s12901-018-0056-1, PMID: 29849503 PMC5968499

[ref95] Hasanvand H. Teymouri F. Ohadi E. Azadegan A. Sadeghi Kalani B. (2019). Biofilm formation in *Staphylococcus epidermidis* isolated from hospitalized patients. Archiv. Clin. Infect. Dis. 14:e64496. doi: 10.5812/archcid.64496

[ref96] Higgins J. P. Thompson S. G. (2002). Quantifying heterogeneity in a meta-analysis. Stat. Med. 21, 1539–1558. doi: 10.1002/sim.1186, PMID: 12111919

[ref97] Hoffmann K. den Heijer C. D. George A. Apfalter P. Maier M. (2015). Prevalence and resistance patterns of commensal *S. aureus* in community-dwelling GP patients and socio-demographic associations. A cross-sectional study in the framework of the APRES-project in Austria. BMC Infect. Dis. 15:213. doi: 10.1186/s12879-015-0949-1, PMID: 25981559 PMC4458027

[ref98] Horvath A. Dobay O. Sahin-Toth J. Juhasz E. Pongracz J. Ivan M. . (2020). Characterisation of antibiotic resistance, virulence, clonality and mortality in MRSA and MSSA bloodstream infections at a tertiary-level hospital in Hungary: a 6-year retrospective study. Ann. Clin. Microbiol. Antimicrob. 19:17. doi: 10.1186/s12941-020-00357-z, PMID: 32381015 PMC7206755

[ref99] Ibadin E. E. Enabulele I. O. Muinah F. (2017). Prevalence of mecA gene among staphylococci from clinical samples of a tertiary hospital in Benin City, Nigeria. Afr. Health Sci. 17, 1000–1010. doi: 10.4314/ahs.v17i4.7, PMID: 29937870 PMC5870281

[ref100] Ikuta K. Swetschinski L. Robles G. Sharara F. Mestrovic T. Gray A. . (2022). Global mortality associated with 33 bacterial pathogens in 2019: a systematic analysis for the global burden of disease study 2019. Lancet 400, 2221–2248. doi: 10.1016/S0140-6736(22)02185-7, PMID: 36423648 PMC9763654

[ref101] Iliya S. Mwangi J. Maathai R. Muriuki M. (2020). Phenotypic analysis and antibiotic susceptibility of methicillin-resistant *Staphylococcus aureus* in Kiambu County, Kenya. J. Infect. Dev. Ctries. 14, 597–605. doi: 10.3855/jidc.12174, PMID: 32683350

[ref102] Islam T. A. B. Shamsuzzaman S. (2015). Prevalence and antimicrobial susceptibility pattern of methicillin-resistant, vancomycin-resistant, and Panton-valentine leukocidin positive *Staphylococcus aureus* in a tertiary care hospital Dhaka, Bangladesh. Tzu Chi Med. J. 27, 10–14. doi: 10.1016/j.tcmj.2014.12.001

[ref103] Javidnia S. Talebi M. Katouli M. Shojaie A. Lari A. R. Pourshafie M. R. (2015). Clonal diversity of meticillin-resistant *staphylococcus aureus* isolated from intensive care unit. Infect. Dis. Clin. Pract. 23, 128–130. doi: 10.1097/IPC.0000000000000230

[ref104] Joachim A. Moyo S. J. Nkinda L. Majigo M. Mmbaga E. Mbembati N. . (2017). Prevalence of methicillin-resistant *Staphylococcus aureus* carriage on admission among patients attending regional hospitals in Dar Es Salaam, Tanzania. BMC. Res. Notes 10:417. doi: 10.1186/s13104-017-2668-8, PMID: 28830510 PMC5568238

[ref105] John B. Mabekoje Oladele O. Aminat H. Saba M. A. Danasabe D. Legbo M. I. . (2023). Occurrence of Staphylococcus associated with urinary tract infections among women attending Ibrahim Badamasi Babangida (IBB) specialist hospital, Minna, Nigeria. Tanzan. J. Health Res. 24, 17–30. doi: 10.4314/thrb.v24i2

[ref106] Juda M. Chudzik-Rzad B. Malm A. (2016). The prevalence of genotypes that determine resistance to macrolides, lincosamides, and streptogramins B compared with spiramycin susceptibility among erythromycin-resistant *Staphylococcus epidermidis*. Mem. Inst. Oswaldo Cruz 111, 155–160. doi: 10.1590/0074-02760150356, PMID: 27008373 PMC4804497

[ref107] Junaidi N. S. S. A. Shakrin N. N. S. M. Huri M. F. D. Kamarudin A. Z. Desa M. N. M. Yunus W. M. Z. W. (2023). Antibiotic resistance and molecular typing of clinical *Staphylococcus aureus* isolates from Malaysian military hospital. Asian Pac J Trop Med 16, 220–231. doi: 10.4103/1995-7645.377743

[ref108] Kahsay A. G. Hagos D. G. Abay G. K. Mezgebo T. A. (2018). Prevalence and antimicrobial susceptibility patterns of methicillin-resistant *Staphylococcus aureus* among janitors of Mekelle university, North Ethiopia. BMC. Res. Notes 11:294. doi: 10.1186/s13104-018-3399-1, PMID: 29751844 PMC5948666

[ref109] Kang S. H. Kim M. K. (2019). Antibiotic sensitivity and resistance of bacteria from odontogenic maxillofacial abscesses. J. Korean Assoc. Oral Maxillofac. Surg. 45, 324–331. doi: 10.5125/jkaoms.2019.45.6.324, PMID: 31966977 PMC6955427

[ref110] Khader K. Thomas A. Jones M. Toth D. Stevens V. Samore M. H. . (2019). Variation and trends in transmission dynamics of methicillin-resistant *Staphylococcus aureus* in veterans affairs hospitals and nursing homes. Epidemics 28:100347. doi: 10.1016/j.epidem.2019.100347, PMID: 31171468 PMC7006838

[ref111] Khan F. Y. Abu-Khattab M. Almaslamani E. A. Hassan A. A. Mohamed S. F. Elbuzdi A. A. . (2017). Acute bacterial meningitis in Qatar: a hospital-based study from 2009 to 2013. Biomed. Res. Int. 2017:2975610. doi: 10.1155/2017/2975610 28785577 PMC5530415

[ref112] Khan S. Singh P. Siddiqui Z. Ansari M. (2015). Pregnancy-associated asymptomatic bacteriuria and drug resistance. J. Taibah Univ. Med. Sci. 10, 340–345. doi: 10.1016/j.jtumed.2015.01.011

[ref113] Khanal A. Sulochan G. C. Gaire A. Khanal A. Estrada R. Ghimire R. . (2021). Methicillin-resistant *Staphylococcus aureus* in Nepal: a systematic review and meta-analysis. Int. J. Infect. Dis. 103, 48–55. doi: 10.1016/j.ijid.2020.11.152, PMID: 33217574

[ref114] Khemiri M. Akrout Alhusain A. Abbassi M. S. El Ghaieb H. Santos Costa S. Belas A. . (2017). Clonal spread of methicillin-resistant *Staphylococcus aureus*-t6065-CC5-SCCmecV-agrII in a Libyan hospital. J. Glob. Antimicrob. Resist. 10, 101–105. doi: 10.1016/j.jgar.2017.04.014, PMID: 28729209

[ref115] Kim H. J. Choi Q. Kwon G. C. Koo S. H. (2020). Molecular epidemiology and virulence factors of methicillin-resistant *Staphylococcus aureus* isolated from patients with bacteremia. J. Clin. Lab. Anal. 34:e23077. doi: 10.1002/jcla.23077, PMID: 31721291 PMC7083439

[ref116] Kong Y. Ye J. Zhou W. Jiang Y. Lin H. Zhang X. . (2018). Prevalence of methicillin-resistant *Staphylococcus aureus* colonisation among healthcare workers at a tertiary care hospital in southeastern China. J. Glob. Antimicrob. Resist. 15, 256–261. doi: 10.1016/j.jgar.2018.08.013 30144635

[ref117] Kpeli G. Darko Otchere I. Lamelas A. Buultjens A. L. Bulach D. Baines S. L. . (2016). Possible healthcare-associated transmission as a cause of secondary infection and population structure of *Staphylococcus aureus* isolates from two wound treatment centres in Ghana. New Microb. New Infect. 13, 92–101. doi: 10.1016/j.nmni.2016.07.001, PMID: 27547406 PMC4983152

[ref118] Kuhn M. Wing J. Weston S. Williams A. Keefer C. Engelhardt A. . (2015). Caret: Classification and regression training. (Version 7.0-1) [Computer software]. Available at: https://github.com/topepo/caret/

[ref119] Kulshrestha N. Ghatak T. Gupta P. Singh M. Agarwal J. Mishra P. (2021). Surveillance of health-care workers hand to detect carriage of multidrug-resistant Staphylococcus spp. in a tertiary care center: An observational study. Med. J. Dr. D.Y. Patil Vidyapeeth 14, 403–408. doi: 10.4103/mjdrdypu.mjdrdypu_372_20

[ref120] Kumar S. Shetty V. A. (2021). Prevalence and susceptibility profiles of methicillin sensitive *Staphylococcus aureus* from community and hospital associated infections. J. Clin. Diagn. Res. 15:5. doi: 10.7860/JCDR/2021/48115.14622

[ref121] Kumar R. A. Thirugnanamani R. Dodeja S. Satish H. S. (2018). Bacterial profile and antibiotic sensitivity in patients with chronic rhinosinusitis undergoing functional endoscopic sinus surgery: a prospective study. Int. J. Clin. Rhinol. 10, 137–141. doi: 10.5005/jp-journals-10013-1325, PMID: 35702834

[ref122] Kurup R. Ansari A. A. (2019). A study to identify bacteriological profile and other risk factors among diabetic and non-diabetic foot ulcer patients in a Guyanese hospital setting. Diabetes Metab. Syndr. 13, 1871–1876. doi: 10.1016/j.dsx.2019.04.024, PMID: 31235108

[ref123] Lan T. Zhang B. Liu J. L. Jia Q. Gao J. Cao L. . (2024). Prevalence and antibiotic resistance patterns of methicillin-resistant *Staphylococcus aureus* (MRSA) in a hospital setting: a retrospective study from 2018 to 2022. Indian J. Microbiol. 64, 1035–1043. doi: 10.1007/s12088-024-01228-3, PMID: 39282164 PMC11399522

[ref124] Larsen J. Petersen A. Sorum M. Stegger M. van Alphen L. Valentiner-Branth P. . (2015). Meticillin-resistant *Staphylococcus aureus* CC398 is an increasing cause of disease in people with no livestock contact in Denmark, 1999 to 2011. Euro Surveill. 20. doi: 10.2807/1560-7917.ES.2015.20.37.30021, PMID: 26535590 PMC4902279

[ref125] Leclercq R. (2002). Mechanisms of resistance to macrolides and lincosamides: nature of the resistance elements and their clinical implications. Clin. Infect. Dis. 34, 482–492. doi: 10.1086/324626, PMID: 11797175

[ref126] Lee Y. C. Chen P. Y. Wang J. T. Chang S. C. (2020). Prevalence of fosfomycin resistance and gene mutations in clinical isolates of methicillin-resistant *Staphylococcus aureus*. Antimicrob. Resist. Infect. Control 9:135. doi: 10.1186/s13756-020-00790-x, PMID: 32807239 PMC7430020

[ref127] Lee S. O. Lee S. Park S. Lee J. E. Lee S. H. (2019). The cefazolin inoculum effect and the presence of type a blaZ gene according to agr genotype in methicillin-susceptible *Staphylococcus aureus* bacteremia. Infect Chemother. 51, 376–385. doi: 10.3947/ic.2019.51.4.376, PMID: 31898425 PMC6940372

[ref128] Leibler J. H. Leon C. Cardoso L. J. P. Morris J. C. Miller N. S. Nguyen D. D. . (2017). Prevalence and risk factors for MRSA nasal colonization among persons experiencing homelessness in Boston, MA. J. Med. Microbiol. 66, 1183–1188. doi: 10.1099/jmm.0.000552, PMID: 28771129 PMC7001487

[ref129] Lenart-Boron A. Wolny-Koladka K. Stec J. Kasprowic A. (2016). Phenotypic and molecular antibiotic resistance determination of airborne coagulase negative Staphylococcus spp. strains from healthcare facilities in southern Poland. Microb. Drug Resist. 22, 515–522. doi: 10.1089/mdr.2015.0271, PMID: 26978225

[ref130] Lennartz F. E. Schwartbeck B. Dubbers A. Grosse-Onnebrink J. Kessler C. Kuster P. . (2019). The prevalence of *Staphylococcus aureus* with mucoid phenotype in the airways of patients with cystic fibrosis-a prospective study. Int. J. Med. Microbiol. 309, 283–287. doi: 10.1016/j.ijmm.2019.05.002, PMID: 31122879

[ref131] Li S. Guo Y. Zhao C. Chen H. Hu B. Chu Y. . (2016). In vitro activities of tedizolid compared with other antibiotics against gram-positive pathogens associated with hospital-acquired pneumonia, skin and soft tissue infection and bloodstream infection collected from 26 hospitals in China. J. Med. Microbiol. 65, 1215–1224. doi: 10.1099/jmm.0.000347, PMID: 27599998

[ref132] Li S. Han Z. He J. Gao S. Liu D. Liu L. . (2018). Society for Translational Medicine expert consensus on the use of antibacterial drugs in thoracic surgery. J. Thorac. Dis. 10, 6356–6374. doi: 10.21037/jtd.2018.10.108, PMID: 30622808 PMC6297397

[ref133] Liang J. Hu Y. Fu M. Li N. Wang F. Yu X. . (2023). Resistance and molecular characteristics of methicillin-resistant Staphylococcus aureus and heterogeneous vancomycin-intermediate *Staphylococcus aureus*. Infect Drug Resist 16, 379–388. doi: 10.2147/IDR.S392908, PMID: 36714350 PMC9882971

[ref134] Liang B. Mai J. Liu Y. Huang Y. Zhong H. Xie Y. . (2018). Prevalence and characterization of *Staphylococcus aureus* isolated from women and children in Guangzhou, China. Front. Microbiol. 9:2790. doi: 10.3389/fmicb.2018.02790, PMID: 30505300 PMC6250813

[ref135] Lin J. Wu C. Yan C. Ou Q. Lin D. Zhou J. . (2018). A prospective cohort study of Staphylococcus aureus and methicillin-resistant *Staphylococcus aureus* carriage in neonates: the role of maternal carriage and phenotypic and molecular characteristics. Infect Drug Resist 11, 555–565. doi: 10.2147/IDR.S157522, PMID: 29731644 PMC5926071

[ref136] Liu C. Chen Z. J. Sun Z. Feng X. Zou M. Cao W. . (2015). Molecular characteristics and virulence factors in methicillin-susceptible, resistant, and heterogeneous vancomycin-intermediate *Staphylococcus aureus* from Central-Southern China. J. Microbiol. Immunol. Infect. 48, 490–496. doi: 10.1016/j.jmii.2014.03.003, PMID: 24767415

[ref137] Livermore D. M. Mushtaq S. Warner M. James D. Kearns A. Woodford N. (2015). Pathogens of skin and skin-structure infections in the UK and their susceptibility to antibiotics, including ceftaroline. J. Antimicrob. Chemother. 70, 2844–2853. doi: 10.1093/jac/dkv179, PMID: 26142478

[ref138] Lodise T. P. Jr. McKinnon P. S. (2007). Burden of methicillin-resistant *Staphylococcus aureus*: focus on clinical and economic outcomes. Pharmacotherapy 27, 1001–1012. doi: 10.1592/phco.27.7.1001, PMID: 17594206

[ref139] Luo Z. G. Ying X. R. Shen C. Ren Y. Wang S. B. Wu G. F. (2020). Characteristics and drug resistance of pathogens in urinary tract infection patients complicated with urinary calculi. Indian J. Pharm. Sci. 82, 922–927.

[ref140] Mahfouz A. A. Said H. S. Elfeky S. M. Shaaban M. I. (2023). Inhibition of erythromycin and erythromycin-induced resistance among *Staphylococcus aureus* clinical isolates. Antibiotics (Basel) 12. doi: 10.3390/antibiotics12030503, PMID: 36978370 PMC10044026

[ref141] Maina D. Omuse G. Revathi G. Adam R. D. (2016). Spectrum of microbial diseases and resistance patterns at a private teaching Hospital in Kenya: implications for clinical practice. PLoS One 11:e0147659. doi: 10.1371/journal.pone.0147659, PMID: 26807811 PMC4726487

[ref142] Maleki D. T. Ghalavand Z. Laabei M. Nikmanesh B. Houri H. Kodori M. . (2019). Molecular analysis of accessory gene regulator functionality and virulence genes in *Staphylococcus aureus* derived from pediatric wound infections. Infect. Genet. Evol. 73, 255–260. doi: 10.1016/j.meegid.2019.05.013, PMID: 31102739

[ref143] Mama M. Aklilu A. Misgna K. Tadesse M. Alemayehu E. (2019). Methicillin- and inducible clindamycin-resistant *Staphylococcus aureus* among patients with wound infection attending Arba Minch hospital, South Ethiopia. Int J Microbiol 2019:2965490. doi: 10.1155/2019/2965490 31065270 PMC6466912

[ref144] Manandhar S. Shrestha R. Tuladhar R. S. Lekhak S. (2021). Inducible clindamycin resistance and biofilm production among staphylococci isolated from tertiary care hospitals in Nepal. Infect Dis. Rep. 13, 1043–1052. doi: 10.3390/idr13040095, PMID: 34940405 PMC8702181

[ref145] Mansson E. Hellmark B. Sundqvist M. Soderquist B. (2015). Sequence types of *Staphylococcus epidermidis* associated with prosthetic joint infections are not present in the laminar airflow during prosthetic joint surgery. APMIS 123, 589–595. doi: 10.1111/apm.12392, PMID: 25951935

[ref146] Mascaro V. Capano M. S. Iona T. Nobile C. G. A. Ammendolia A. Pavia M. (2019). Prevalence of *Staphylococcus aureus* carriage and pattern of antibiotic resistance, including methicillin resistance, among contact sport athletes in Italy. Infect Drug Resist. 12, 1161–1170. doi: 10.2147/IDR.S195749, PMID: 31123413 PMC6511236

[ref147] McHardy I. H. Veltman J. Hindler J. Bruxvoort K. Carvalho M. M. Humphries R. M. (2017). Clinical and microbiological aspects of beta-lactam resistance in *Staphylococcus lugdunensis*. J. Clin. Microbiol. 55, 585–595. doi: 10.1128/JCM.02092-16, PMID: 27927926 PMC5277529

[ref148] Mehreen A. Liaqat I. Arshad M. Waheed M. Arshad N. (2018). Characterization of *Staphylococcus aureus* from sore throat patients: association among host immune evasion and toxin genes. Pak. J. Zool. 50. doi: 10.17582/journal.pjz/2018.50.6.2261.2272

[ref149] Mesbah Elkammoshi A. Ghasemzadeh-Moghaddam H. Amin Nordin S. Mohd Taib N. Kumar Subbiah S. Neela V. . (2016). A low prevalence of inducible macrolide, Lincosamide, and Streptogramin B resistance phenotype among methicillin-susceptible *Staphylococcus aureus* isolated from Malaysian patients and healthy individuals. Jundishapur J. Microbiol. 9:e37148, PMID: 27942364 10.5812/jjm.37148 PMC5136447

[ref150] Miklasinska-Majdanik M. (2021). Mechanisms of resistance to macrolide antibiotics among *Staphylococcus aureus*. Antibiotics (Basel) 10. doi: 10.3390/antibiotics10111406, PMID: 34827344 PMC8615237

[ref151] Modukuru G. K. Surya P. M. S. Kakumanu V. R. Yarava S. (2021). Phenotypic characterization of macrolide-Lincosamide-Streptogramin B resistance in *Staphylococcus aureus*. J. Pure Appl. Microbiol. 15, 689–694. doi: 10.22207/JPAM.15.2.18

[ref152] Mostafa M. Siadat S. D. Shahcheraghi F. Vaziri F. Japoni-Nejad A. Vand Yousefi J. . (2015). Variability in gene cassette patterns of class 1 and 2 integrons associated with multi drug resistance patterns in *Staphylococcus aureus* clinical isolates in Tehran-Iran. BMC Microbiol. 15:152. doi: 10.1186/s12866-015-0488-3, PMID: 26228695 PMC4521504

[ref153] Mottola C. Matias C. S. Mendes J. J. Melo-Cristino J. Tavares L. Cavaco-Silva P. . (2016). Susceptibility patterns of *Staphylococcus aureus* biofilms in diabetic foot infections. BMC Microbiol. 16:119. doi: 10.1186/s12866-016-0737-0, PMID: 27339028 PMC4918071

[ref154] Muhammad A. Khan S. N. Ali N. Rehman M. U. Ali I. (2020). Prevalence and antibiotic susceptibility pattern of uropathogens in outpatients at a tertiary care hospital. New Microbes New Infect 36:100716. doi: 10.1016/j.nmni.2020.100716, PMID: 32637123 PMC7330609

[ref155] Murugesan S. Perumal N. Mahalingam S. P. Dilliappan S. K. Krishnan P. (2015). Analysis of antibiotic resistance genes and its associated SCCmec types among nasal carriage of methicillin resistant coagulase negative staphylococci from community settings, Chennai, southern India. J. Clin. Diagn. Res. 9:DC01-05. doi: 10.7860/JCDR/2015/11733.6307, PMID: 26435940 PMC4576531

[ref156] Mutonga D. M. Mureithi M. W. Ngugi N. N. Otieno F. C. F. (2019). Bacterial isolation and antibiotic susceptibility from diabetic foot ulcers in Kenya using microbiological tests and comparison with RT-PCR in detection of S. Aureus and MRSA. BMC. Res. Notes 12:244. doi: 10.1186/s13104-019-4278-0, PMID: 31036061 PMC6489269

[ref157] Naghavi M. Vollset S. Ikuta K. Swetschinski L. Gray A. Wool E. . (2024). Global burden of bacterial antimicrobial resistance 1990-2021: a systematic analysis with forecasts to 2050. Lancet 404, 1199–1226. doi: 10.1016/S0140-6736(24)01867-1, PMID: 39299261 PMC11718157

[ref158] Napp M. Daeschlein G. von Podewils S. Hinz P. Emmert S. Haase H. . (2016). In vitro susceptibility of methicillin-resistant and methicillin-susceptible strains of *Staphylococcus aureus* to two different cold at plasma sources. Infection 44, 531–537. doi: 10.1007/s15010-016-0888-9, PMID: 26951157

[ref159] Nasirian S. Saadatmand S. Goudarzi H. Goudarzi M. Azimi H. (2018). Molecular investigation of methicillin-resistant *Staphylococcus aureus* strains recovered from the intensive care unit (ICU) based on toxin, adhesion genes and agr locus type analysis. Arch. Clin. Infect. Dis. 13:e14495.

[ref160] Nichol K. A. Adam H. J. Golding G. R. Lagace-Wiens P. R. S. Karlowsky J. A. Hoban D. J. . (2019). Characterization of MRSA in Canada from 2007 to 2016. J. Antimicrob. Chemother. 74, iv55–iv63. doi: 10.1093/jac/dkz288, PMID: 31505646

[ref161] Noordin A. Sapri H. F. Mohamad Sani N. A. Leong S. K. Tan X. E. Tan T. L. . (2016). Antimicrobial resistance profiling and molecular typing of methicillin-resistant *Staphylococcus aureus* isolated from a Malaysian teaching hospital. J. Med. Microbiol. 65, 1476–1481. doi: 10.1099/jmm.0.000387, PMID: 27902380

[ref162] Numanovic F. Dermota U. Smajlovic J. Janezic S. Tihic N. Delibegovic Z. . (2021). Characterization and clonal representation of MRSA strains in Tuzla Canton, Bosnia and Herzegovina, from 2009 to 2017. Med. Glas. (Zenica) 18, 38–46. doi: 10.17392/1265-21, PMID: 33345531

[ref163] Okuda K. V. Toepfner N. Alabi A. S. Arnold B. Belard S. Falke U. . (2016). Molecular epidemiology of *Staphylococcus aureus* from Lambarene, Gabon. Eur. J. Clin. Microbiol. Infect. Dis. 35, 1963–1973. doi: 10.1007/s10096-016-2748-z, PMID: 27553495

[ref164] Olufunmiso O. Tolulope I. Roger C. (2017). Multidrug and vancomycin resistance among clinical isolates of *Staphylococcus aureus* from different teaching hospitals in Nigeria. Afr. Health Sci. 17, 797–807. doi: 10.4314/ahs.v17i3.23, PMID: 29085408 PMC5656204

[ref165] Ouidri M. A. (2018). Screening of nasal carriage of methicillin-resistant *Staphylococcus aureus* during admission of patients to Frantz fanon hospital, Blida, Algeria. New Microbes New Infect 23, 52–60. doi: 10.1016/j.nmni.2018.02.006, PMID: 29692907 PMC5913062

[ref166] Oydanich M. Dingle T. C. Hamula C. L. Ghisa C. Asbell P. (2017). Retrospective report of antimicrobial susceptibility observed in bacterial pathogens isolated from ocular samples at Mount Sinai hospital, 2010 to 2015. Antimicrob. Resist. Infect. Control 6:29. doi: 10.1186/s13756-017-0185-0, PMID: 28344783 PMC5360068

[ref167] Parastan R. Kargar M. Solhjoo K. Kafilzadeh F. (2020). A synergistic association between adhesion-related genes and multidrug resistance patterns of *Staphylococcus aureus* isolates from different patients and healthy individuals. J. Glob. Antimicrob. Resist. 22, 379–385. doi: 10.1016/j.jgar.2020.02.025, PMID: 32169685

[ref168] Peng X. Zhu Q. Liu J. Zeng M. Qiu Y. Zhu C. . (2021). Prevalence and antimicrobial resistance patterns of bacteria isolated from cerebrospinal fluid among children with bacterial meningitis in China from 2016 to 2018: a multicenter retrospective study. Antimicrob. Resist. Infect. Control 10:24. doi: 10.1186/s13756-021-00895-x 33516275 PMC7847565

[ref169] Peterside O. Pondei K. Akinbami F. O. (2015). Bacteriological profile and antibiotic susceptibility pattern of neonatal Sepsis at a teaching Hospital in Bayelsa State, Nigeria. Trop. Med. Health 43, 183–190. doi: 10.2149/tmh.2015-03, PMID: 26543394 PMC4593775

[ref170] Petrović J. L. Kuljić K. N. Ristanović E. Jošić D. Lepšanović Z. (2016). Prevalence of Panton-valentine leukocidin genes in community-associated methicillin-resistant *Staphylococcus aureus* in the district of Pomoravlje. Vojnosanit. Pregl. 73, 256–260. doi: 10.2298/VSP140715003P, PMID: 27295910

[ref171] Pfaller M. A. Huband M. D. Shortridge D. Flamm R. K. (2020). Surveillance of Omadacycline activity tested against clinical isolates from the United States and Europe: report from the SENTRY antimicrobial surveillance program, 2016 to 2018. Antimicrob. Agents Chemother. 64:e02488–19. doi: 10.1128/AAC.02488-19, PMID: 32071045 PMC7179604

[ref172] Pradhan P. Rajbhandari P. Nagaraja S. B. Shrestha P. Grigoryan R. Satyanarayana S. . (2021). Prevalence of methicillin-resistant *Staphylococcus aureus* in a tertiary hospital in Nepal. Public Health Action 11, 46–51. doi: 10.5588/pha.21.0042, PMID: 34778015 PMC8575383

[ref173] Preeja P. P. Kumar S. H. Shetty V. (2021). Prevalence and characterization of methicillin-resistant *Staphylococcus aureus* from community- and hospital-associated infections: a tertiary care center study. Antibiotics (Basel) 10:197. doi: 10.3390/antibiotics10020197, PMID: 33670648 PMC7922968

[ref174] Pushkar Aashana Sharma M. Yadav A. (2022). "Prevalence and antimicrobial resistance of methicillin resistant *Staphylococcus aureus* (MRSA) isolated from blood culture in tertiary care hospital in Haryana." Pravara. Med. Rev. 14, 64–68. doi: 10.36848/PMR/2022/99100.51095

[ref175] Qin Y. Wen F. Zheng Y. Zhao R. Hu Q. Zhang R. (2017). Antimicrobial resistance and molecular characteristics of methicillin-resistant *Staphylococcus aureus* isolates from child patients of high-risk wards in Shenzhen, China. Jpn. J. Infect. Dis. 70, 479–484. doi: 10.7883/yoken.JJID.2016.328, PMID: 28250256

[ref176] Rahimi F. (2016). Characterization of resistance to aminoglycosides in methicillin-resistant *Staphylococcus aureus* strains isolated from a tertiary Care Hospital in Tehran, Iran. Jundishapur J. Microbiol. 9:e29237. doi: 10.5812/jjm.29237, PMID: 27099687 PMC4833945

[ref177] Rajkumar S. Sistla S. Manoharan M. Sugumar M. Nagasundaram N. Parija S. C. . (2017). Prevalence and genetic mechanisms of antimicrobial resistance in Staphylococcus species: a multicentre report of the indian council of medical research antimicrobial resistance surveillance network. Indian J. Med. Microbiol. 35, 53–60. doi: 10.4103/ijmm.IJMM_16_427, PMID: 28303819

[ref178] Ramakrishna M. S. Jeyamani L. Abimannan G. C. Vajravelu L. K. (2021). Microbial profile and Antibiogram pattern analysis of skin and soft tissue infections at a tertiary Care Center in South India. J. Pure Appl. Microbiol. 15, 915–925. doi: 10.22207/JPAM.15.2.50

[ref179] Rampelotto R. F. Coelho S. S. Franco L. N. Mota A. D. D. Calegari L. F. Jacobi L. F. . (2022). Coagulase-negative staphylococci isolates from blood cultures of newborns in a tertiary hospital in southern Brazil. Braz. J. Pharm. Sci. 58:e19664. doi: 10.1590/s2175-97902022e19664

[ref180] Raut S. Bajracharya K. Adhikari J. Pant S. S. Adhikari B. (2017). Prevalence of methicillin resistant *Staphylococcus aureus* in Lumbini medical college and teaching hospital, Palpa, Western Nepal. BMC. Res. Notes 10:187. doi: 10.1186/s13104-017-2515-y, PMID: 28577365 PMC5457603

[ref181] Roden L. Gorlich D. Omran H. Peters G. Grosse-Onnebrink J. Kahl B. C. (2019). A retrospective analysis of the pathogens in the airways of patients with primary ciliary dyskinesia. Respir. Med. 156, 69–77. doi: 10.1016/j.rmed.2019.08.009, PMID: 31437650

[ref182] Rukan M. Jamil H. Bokhari H. A. Khattak A. A. Khan A. N. Ullah Z. . (2021). Nasal carriage of highly resistant methicillin resistant *Staphylococcus aureus* (MRSA) strains by hospital staff in Hazara region of Pakistan. J. Pak. Med. Assoc. 71, 47–50. doi: 10.47391/JPMA.177, PMID: 33484517

[ref183] Saini V. Jain C. Singh N. P. Alsulimani A. Gupta C. Dar S. A. . (2021). Paradigm shift in antimicrobial resistance pattern of bacterial isolates during the COVID-19 pandemic. Antibiotics (Basel) 10:954. doi: 10.3390/antibiotics10080954, PMID: 34439004 PMC8388877

[ref184] Sakabe D. Del Fiol Fde S. (2016). Profile of infections and antimicrobial treatment among burn-injury patients. Am. J. Infect. Control 44, 950–952. doi: 10.1016/j.ajic.2016.03.063, PMID: 27324611

[ref185] Salah A. Al-Subol I. Hudna A. Alhaj A. Alqubaty A. R. Farie W. . (2021). Neonatal sepsis in Sana'a city, Yemen: a predominance of *Burkholderia cepacia*. BMC Infect. Dis. 21:1108. doi: 10.1186/s12879-021-06808-y, PMID: 34706677 PMC8554861

[ref186] Salarvand S. Abdollahi A. Doraghi M. Miratashi Yazdi S. A. Panahi Z. Mortazavi S. M. J. . (2023). Microbiological profile and drug resistance in bone and joint infections: a survey in orthopedic wards of a great referral Hospital in Tehran, Iran. Jundishapur J. Microbiol. 16:e137125. doi: 10.5812/jjm-137125

[ref187] Saleem M. Ahmad I. Salem A. M. Almarshedy S. M. Moursi S. A. Syed Khaja A. S. . (2025). Molecular and genetic analysis of methicillin-resistant *Staphylococcus aureus* (MRSA) in a tertiary care hospital in Saudi Arabia. Naunyn. Schmiedebergs Arch. Pharmacol. doi: 10.1007/s00210-024-03771-8, PMID: 39777537

[ref188] Sanchez A. Benito N. Rivera A. Garcia L. Miro E. Mur I. . (2020). Pathogenesis of *Staphylococcus epidermidis* in prosthetic joint infections: can identification of virulence genes differentiate between infecting and commensal strains? J. Hosp. Infect. 105, 561–568. doi: 10.1016/j.jhin.2020.04.026, PMID: 32339618

[ref189] Sapkota J. Sharma M. Jha B. Bhatt C. P. (2019). Prevalence of *Staphylococcus aureus* isolated from clinical samples in a tertiary care hospital: a descriptive cross-sectional study. JNMA J. Nepal Med. Assoc. 57, 398–402. doi: 10.31729/jnma.4673, PMID: 32335648 PMC7580409

[ref190] Saxena S. Priyadarshi M. Saxena A. Singh R. (2019). Antimicrobial consumption and bacterial resistance pattern in patients admitted in I.C.U at a tertiary care center. J. Infect. Public Health 12, 695–699. doi: 10.1016/j.jiph.2019.03.014, PMID: 31000490

[ref191] Selim S. Faried O. A. Almuhayawi M. S. Saleh F. M. Sharaf M. El Nahhas N. . (2022). Incidence of vancomycin-resistant *Staphylococcus aureus* strains among patients with urinary tract infections. Antibiotics (Basel) 11:408. doi: 10.3390/antibiotics11030408, PMID: 35326871 PMC8944512

[ref192] Shashindran N. Nagasundaram N. Thappa D. M. Sistla S. (2016). Can Panton valentine Leukocidin gene and clindamycin susceptibility serve as predictors of community origin of MRSA from skin and soft tissue infections? J. Clin. Diagn. Res. 10, DC01–DC04. doi: 10.7860/JCDR/2016/14531.7036, PMID: 26894063 PMC4740590

[ref193] Sheeba V. Vedachalam D. Affan T. F. (2021). An increasing trend in the antimicrobial resistance of bacterial isolates from skin and soft tissue infections in a tertiary care hospital. J. Pure Appl. Microbiol. 15, 803–812. doi: 10.22207/JPAM.15.2.34

[ref194] Shidiki A. Pandit B. Vyas A. (2018). Incidence and antibiotic profile of bacterial isolates from neonatal septicemia in national medical college and teaching hospital, Birgunj, Nepal. Res. J. Pharm. Technol. 11, 2238–2242. doi: 10.5958/0974-360X.2018.00414.6

[ref195] Shittu A. O. Oyedara O. Okon K. Raji A. Peters G. von Muller L. . (2015). An assessment on DNA microarray and sequence-based methods for the characterization of methicillin-susceptible *Staphylococcus aureus* from Nigeria. Front. Microbiol. 6:1160. doi: 10.3389/fmicb.2015.01160 26539185 PMC4612102

[ref196] Shivappa S. G. Morubagal R. R. Mahale R. P. Gowda R. S. (2018). Prevalence and Antibiogram of methicillin sensitive and methicillin resistant *Staphylococcus aureus* isolated from pus samples in a tertiary care teaching hospital. J. Pure Appl. Microbiol. 12, 2297–2303. doi: 10.22207/JPAM.12.4.71

[ref197] Singh N. Hota S., S. snigdha Panda, D. Pattnaik, A. Praharaj and J. Jena (2019). "Prevalence and antibiotic resistance profile of coagulase negative staphylococci causing true Bacteraemia in a tertiary care hospital." Indian J. Public Health 10:479. doi: 10.5958/0976-5506.2019.02474.4

[ref198] Skender K. Machowska A. Singh V. Goel V. Marothi Y. Lundborg C. S. . (2022). Antibiotic use, incidence and risk factors for orthopedic surgical site infections in a teaching Hospital in Madhya Pradesh, India. Antibiotics (Basel) 11:748. doi: 10.3390/antibiotics11060748, PMID: 35740154 PMC9220190

[ref199] Solomon J. G. Salaudeen A. G. (2021). Antibiotics resistance, sensitivity pattern and development of antibiogram to support empirical prescription in health facilities in south senatorial district of Kwara state, Nigeria 9, 35–45. doi: 10.21522/TIJPH.2013.09.03.Art004

[ref200] Soroush S. Jabalameli F. Taherikalani M. Amirmozafari N. Fooladi A. A. Asadollahi K. . (2016). Investigation of biofilm formation ability, antimicrobial resistance and the staphylococcal cassette chromosome mec patterns of methicillin resistant *Staphylococcus epidermidis* with different sequence types isolated from children. Microb. Pathog. 93, 126–130. doi: 10.1016/j.micpath.2016.01.018, PMID: 26821355

[ref201] Sotoudeh Anvari M. Kianinejad R. Boroumand M. A. Arzhan S. Jalali A. (2015). Bacterial pericarditis and antimicrobial resistance at the Tehran Heart Center, Iran. J. Infect. Dev. Ctries. 9, 780–784. doi: 10.3855/jidc.6027, PMID: 26230130

[ref202] Soumya K. R. Philip S. Sugathan S. Mathew J. Radhakrishnan E. K. (2017). Virulence factors associated with Coagulase Negative Staphylococci isolated from human infections. 3 Biotech 7:140. doi: 10.1007/s13205-017-0753-2 PMC5462657 28593524

[ref203] Sterne J. A. C. Egger M. (2005). Regression methods to detect publication and other Bias in Meta-analysis. Public. Bias Meta Analysis, 99–110. doi: 10.1002/0470870168.ch6

[ref204] Sultan A. Rizvi M. Khan F. Sami H. Shukla I. Khan H. M. (2015). Increasing antimicrobial resistance among uropathogens: is fosfomycin the answer? Urol. Ann. 7, 26–30. doi: 10.4103/0974-7796.148585, PMID: 25657539 PMC4310112

[ref205] Suneel Kumar A. Smiline Girija A. S. Naga Srilatha B. (2021). Characterization of biofilm producing methicillin resistant coagulase negative staphylococci from India. Acta Microbiol. Immunol. Hung. 69, 35–40. doi: 10.1556/030.2021.01538 34898472

[ref206] Sutter D. E. Milburn E. Chukwuma U. Dzialowy N. Maranich A. M. Hospenthal D. R. (2016). Changing susceptibility of *Staphylococcus aureus* in a US pediatric population. Pediatrics 137: e20153099. doi: 10.1542/peds.2015-3099, PMID: 26933211

[ref207] Svent-Kucina N. Pirs M. Kofol R. Blagus R. Smrke D. M. Bilban M. . (2016). Molecular characterization of *Staphylococcus aureus* isolates from skin and soft tissue infections samples and healthy carriers in the Central Slovenia region. APMIS 124, 309–318. doi: 10.1111/apm.12509, PMID: 26781044

[ref208] Taha L. Stegger M. Soderquist B. (2019). *Staphylococcus lugdunensis*: antimicrobial susceptibility and optimal treatment options. Eur. J. Clin. Microbiol. Infect. Dis. 38, 1449–1455. doi: 10.1007/s10096-019-03571-6, PMID: 31144243 PMC6647525

[ref209] Tahbaz S. V. Azimi L. Nowroozi J. Armin S. Fallah F. (2019). Multilocus sequence typing and antibiotic resistant patterns of the meticillin-resistant *Staphylococcus aureus* isolates from different clinical specimens. Rev. Res. Med. Microbiol. 30, 77–82. doi: 10.1097/MRM.0000000000000176

[ref210] Taherirad A. Jahanbakhsh R. Shakeri F. Anvary S. Ghaemi E. A. (2016). Staphylococcal cassette chromosome mec types among methicillin-resistant *Staphylococcus aureus* in northern Iran. Jundishapur J. Microbiol. 9:e33933, PMID: 27800133 10.5812/jjm.33933 PMC5080676

[ref211] Talapan D. Sandu A. M. Rafila A. (2023). Antimicrobial resistance of *Staphylococcus aureus* isolated between 2017 and 2022 from infections at a tertiary Care Hospital in Romania. Antibiotics (Basel) 12:974. doi: 10.3390/antibiotics12060974, PMID: 37370293 PMC10294969

[ref212] Tang B. Gong T. Cui Y. Wang L. He C. Lu M. . (2020). Characteristics of oral methicillin-resistant *Staphylococcus epidermidis* isolated from dental plaque. Int. J. Oral Sci. 12:15. doi: 10.1038/s41368-020-0079-5, PMID: 32385260 PMC7210960

[ref213] Tekeli A. Ocal D. N. Ozmen B. B. Karahan Z. C. Dolapci I. (2016). Molecular characterization of methicillin-resistant *Staphylococcus aureus* bloodstream isolates in a Turkish university hospital between 2002 and 2012. Microb. Drug Resist. 22, 564–569. doi: 10.1089/mdr.2015.0116, PMID: 26982281

[ref214] Tong S. Y. Davis J. S. Eichenberger E. Holland T. L. Fowler V. G. Jr. (2015). *Staphylococcus aureus* infections: epidemiology, pathophysiology, clinical manifestations, and management. Clin. Microbiol. Rev. 28, 603–661. doi: 10.1128/CMR.00134-14, PMID: 26016486 PMC4451395

[ref215] Tsige Y. Tadesse S. Eyesus T. Tefera M. M. Amsalu A. Menberu M. A. (2020). Prevalence of methicillin-resistant Staphylococcus aureus and associated risk factors among patients with wound infection at referral hospital, Northeast Ethiopia. J Pathog 2020:3168325. 32566311 10.1155/2020/3168325 PMC7271240

[ref216] Ukpai E. G. Chukwura E. I. Moses I. B. Ugbo E. N. Agumah N. B. Okata-Nwali O. D. . (2021). Prevalence and Antibiogram of healthcare-associated methicillin-resistant *Staphylococcus aureus* (HA-MRSA) in Ebonyi state, Nigeria. Int. J. Pharma. Sci. Rev. Res. 69, 104–111. doi: 10.47583/ijpsrr.2021.v69i01.016

[ref217] Ullah H. Bashir K. Idrees M. Ullah A. Hassan N. Khan S. . (2022). Phylogenetic analysis and antimicrobial susceptibility profile of uropathogens. PLoS One 17:e0262952. doi: 10.1371/journal.pone.0262952, PMID: 35089940 PMC8797202

[ref219] Uyar Güleç G. Öncü S. Bozdoğan B. Öztürk B. Ertuğrul B. Sakarya S. (2020). Phenotypic and molecular detection of macrolide lincosamide streptogramin B resistance in clinical isolates of staphylococci. FLORA 25, 190–196. doi: 10.5578/flora.68683

[ref218] Valle D. L. Paclibare P. A. Cabrera E. C. Rivera W. L. (2016). Molecular and phenotypic characterization of methicillin-resistant *Staphylococcus aureus* isolates from a tertiary hospital in the Philippines. Trop Med Health 44:3. doi: 10.1186/s41182-016-0003-z, PMID: 27398062 PMC4934148

[ref220] Viechtbauer W. (2010). Conducting Meta-analyses in R with the meta for Package. J. Stat. Softw. 36, 1–48. doi: 10.18637/jss.v036.i03, PMID: 39902325

[ref221] Viechtbauer W. Cheung M. W. (2010). Outlier and influence diagnostics for meta-analysis. Res. Synth. Methods 1, 112–125. doi: 10.1002/jrsm.11, PMID: 26061377

[ref222] Vijay S. Dalela G. (2016). Prevalence of LRTI in patients presenting with productive cough and their antibiotic resistance pattern. J. Clin. Diagn. Res. 10, DC09–DC12. doi: 10.7860/JCDR/2016/17855.7082, PMID: 26894065 PMC4740592

[ref223] Wan T. W. Hung W. C. Tsai J. C. Lin Y. T. Lee H. Hsueh P. R. . (2016). Novel structure of *Enterococcus faecium*-originated ermB-positive Tn1546-like element in *Staphylococcus aureus*. Antimicrob. Agents Chemother. 60, 6108–6114. doi: 10.1128/AAC.01096-16, PMID: 27480862 PMC5038305

[ref224] Wang R. Li X. Wang Q. Zhang Y. Wang H. (2017). Microbiological characteristics and clinical features of cardiac implantable electronic device infections at a tertiary Hospital in China. Front. Microbiol. 8:360. doi: 10.3389/fmicb.2017.00360 28321212 PMC5337500

[ref225] Wangai F. K. Masika M. M. Lule G. N. Karari E. M. Maritim M. C. Jaoko W. G. . (2019). Bridging antimicrobial resistance knowledge gaps: the east African perspective on a global problem. PLoS One 14:e0212131. doi: 10.1371/journal.pone.0212131, PMID: 30742669 PMC6370290

[ref226] Washington J. A. Wilson W. R. (1985). Erythromycin: a microbial and clinical perspective after 30 years of clinical use (1). Mayo Clin Proc. 1985 60:189–203. doi: 10.1016/s0025-6196(12)60219-5 3974301

[ref227] Weldu Y. Naizgi M. Hadgu A. Desta A. A. Kahsay A. Negash L. . (2020). Neonatal septicemia at intensive care unit, Ayder comprehensive specialized hospital, Tigray, North Ethiopia: bacteriological profile, drug susceptibility pattern, and associated factors. PLoS One 15:e0235391. doi: 10.1371/journal.pone.0235391, PMID: 32603368 PMC7326223

[ref228] Wurster J. I. Bispo P. J. M. Van Tyne D. Cadorette J. J. Boody R. Gilmore M. S. (2018). *Staphylococcus aureus* from ocular and otolaryngology infections are frequently resistant to clinically important antibiotics and are associated with lineages of community and hospital origins. PLoS One 13:e0208518. doi: 10.1371/journal.pone.0208518, PMID: 30521630 PMC6283574

[ref229] Xie X. Bao Y. Ouyang N. Dai X. Pan K. Chen B. . (2016). Molecular epidemiology and characteristic of virulence gene of community-acquired and hospital-acquired methicillin-resistant *Staphylococcus aureus* isolates in Sun Yat-sen memorial hospital, Guangzhou, southern China. BMC Infect. Dis. 16:339. doi: 10.1186/s12879-016-1684-y, PMID: 27450316 PMC4957337

[ref230] Xu Z. Liu S. Chen L. Liu Y. Tan L. Shen J. . (2019). Antimicrobial resistance and molecular characterization of methicillin-resistant coagulase-negative staphylococci from public shared bicycles in Tianjin, China. J. Glob. Antimicrob. Resist 19, 231–235. doi: 10.1016/j.jgar.2019.03.008, PMID: 30910743

[ref231] Xu X. Zhang X. Zhang G. Abbasi Tadi D. (2024). Prevalence of antibiotic resistance of *Staphylococcus aureus* in cystic fibrosis infection: a systematic review and meta-analysis. J. Glob. Antimicrob. Resist. 36, 419–425. doi: 10.1016/j.jgar.2023.05.006, PMID: 37211214

[ref232] Yadav S. Kapley A. (2021). Antibiotic resistance: global health crisis and metagenomics. Biotechnol. Rep. (Amst.) 29:e00604. doi: 10.1016/j.btre.2021.e00604 33732632 PMC7937537

[ref233] Yang H. Wang W. S. Tan Y. Zhang D. J. Wu J. J. Lei X. (2017). Investigation and analysis of the characteristics and drug sensitivity of bacteria in skin ulcer infections. Chin. J. Traumatol. 20, 194–197. doi: 10.1016/j.cjtee.2016.09.005, PMID: 28689800 PMC5555241

[ref234] Yao Z. Wu Y. Xu H. Lei Y. Long W. Li M. . (2023). Prevalence and clinical characteristics of methicillin-resistant *Staphylococcus aureus* infections among dermatology inpatients: a 7-year retrospective study at a tertiary care center in Southwest China. Front. Public Health 11:1124930. doi: 10.3389/fpubh.2023.1124930, PMID: 36998271 PMC10043400

[ref235] Yitayeh L. Gize A. Kassa M. Neway M. Afework A. Kibret M. . (2021). Antibiogram profiles of Bacteria isolated from different body site infections among patients admitted to GAMBY teaching general hospital, Northwest Ethiopia. Infect Drug Resist 14, 2225–2232. doi: 10.2147/IDR.S307267, PMID: 34163187 PMC8214533

[ref236] Yu W. Kim H. K. Rauch S. Schneewind O. Missiakas D. (2017). Pathogenic conversion of coagulase-negative staphylococci. Microbes Infect. 19, 101–109. doi: 10.1016/j.micinf.2016.12.002, PMID: 28012900 PMC5274588

[ref237] Zamanian M. H. Shirvani M. Janbakhsh A. Sayad B. Vaziri S. Mohseni Afshar Z. . (2021). Antibiotic Resistance in *Staphylococcus aureus* in Patients Hospitalized in Imam Reza Hospital of Kermanshah, Iran (2016–2018). J. Kermanshah Univ. Med. Sci. 25:e118807.

[ref238] Zhang J. Gu F. F. Zhao S. Y. Xiao S. Z. Wang Y. C. Guo X. K. . (2015). Prevalence and molecular epidemiology of *Staphylococcus aureus* among residents of seven nursing homes in Shanghai. PLoS One 10:e0137593. doi: 10.1371/journal.pone.0137593, PMID: 26340648 PMC4560451

[ref239] Zhou K. Sun F. Xu X. L. Hao X. K. Liu J. Y. (2020). Prevalences and characteristics of cultivable nasal bacteria isolated from preclinical medical students. J. Int. Med. Res. 48:300060520961716. doi: 10.1177/0300060520961716, PMID: 33103543 PMC7607144

